# Predicting gastric cancer outcome from resected lymph node histopathology images using deep learning

**DOI:** 10.1038/s41467-021-21674-7

**Published:** 2021-03-12

**Authors:** Xiaodong Wang, Ying Chen, Yunshu Gao, Huiqing Zhang, Zehui Guan, Zhou Dong, Yuxuan Zheng, Jiarui Jiang, Haoqing Yang, Liming Wang, Xianming Huang, Lirong Ai, Wenlong Yu, Hongwei Li, Changsheng Dong, Zhou Zhou, Xiyang Liu, Guanzhen Yu

**Affiliations:** 1 School of Computer Science and Technology, Xidian University, Xi’an, China; 2 Department of Pathology Center of Gastroenterology, Changhai Hospital, Shanghai, China; 3 Department of Oncology, General Hospital of PLA, Beijing, China; 4 Department of Gastrointestinal Medical Oncology, Jiangxi Provincial Cancer Hospital, Nangchang, China; 5 School of Computer Science, Northwestern Polytechnical University, Xi’an, China; 6 Department of Surgery Oncology, Eastern Hepatobiliary Surgery Hospital, Shanghai, China; 7 Department of Oncology, Longhua Hospital Affiliated to Shanghai University of Traditional Chinese Medicine, Shanghai, China; 8 Shanghai Key Laboratory of Multidimensional Information Processing, East China Normal University, Shanghai, China

**Keywords:** Gastrointestinal cancer, Metastasis, Machine learning

## Abstract

N-staging is a determining factor for prognostic assessment and decision-making for stage-based cancer therapeutic strategies. Visual inspection of whole-slides of intact lymph nodes is currently the main method used by pathologists to calculate the number of metastatic lymph nodes (MLNs). Moreover, even at the same N stage, the outcome of patients varies dramatically. Here, we propose a deep-learning framework for analyzing lymph node whole-slide images (WSIs) to identify lymph nodes and tumor regions, and then to uncover tumor-area-to-MLN-area ratio (T/MLN). After training, our model’s tumor detection performance was comparable to that of experienced pathologists and achieved similar performance on two independent gastric cancer validation cohorts. Further, we demonstrate that T/MLN is an interpretable independent prognostic factor. These findings indicate that deep-learning models could assist not only pathologists in detecting lymph nodes with metastases but also oncologists in exploring new prognostic factors, especially those that are difficult to calculate manually.

## Abstract (web summary)

The ratio of tumour area to metastatic lymph node area (T/MLN) is a clinical indicator that can improve prognosis prediction of gastric cancer. Here, the authors use machine learning on whole slide images to generate a method that can predict metastatic lymph nodes and obtain T/MLN.

## Introduction

Gastric cancer (GC) is the second leading cause of cancer-related death worldwide^1^ and remains one of the most common malignant tumors in Asia^2^. The American Joint Committee on Cancer (AJCC) TNM (tumor node metastasis) staging system is a determining factor for prognostic assessment and decision-making for stage-based therapeutic strategies. This system has been revised several times in order to improve its predictive power over past three decades based on detailed analyses of ongoing large international databases. The 7th and 8th editions of the TNM staging system have become regarded as the best for prognostic prediction and have superior reproducibility as compared to previous iterations of the TNM staging system^3^. In the evolution of these editions, one of the most significant updates was to lymph node (LN) staging. The evidence used in the AJCC N-staging system is based on the number of metastatic LNs (MLNs) observed. N-staging itself, however, is an independent factor in predicting the overall survival of patients with gastric cancer^4^. Moreover, even at the same N stage, the outcome of gastric cancer patients may vary dramatically.

In a routine clinical workflow for diagnosing LN metastases, an intact LN is collected, formalin-fixed, paraffin-embedded, sectioned, and then stained with hematoxylin and eosin (H&E). Under an optical microscope, slides of all dissected lymph nodes are then examined for morphology by a pathologist, who assesses the status of each lymph node and the total number of lymph nodes on each individual’s slides. This process is time-consuming and might be easily misdiagnosed by a pathologist alone due to habituation^5^. One question is that the number of LNs acquired may be less than the number required for prognosis because of technical problems, leading to imprecise N-staging. To resolve this problem, the ratio of MLNs was introduced as an adjunct to N-staging; however, it was not shown to be superior to AJCC N-staging^6^. Another question is that visual examination is considered accurate in cases with high metastatic areas, but is inaccurate in cases with micrometastases due to inter- and intraobserver variability. After central pathology review of the breast cancer patients with originally diagnosed as pN0, 18% were restaged as pN0(i+), 3% as pN1mi, and 0.5% as pN1+^5^. Moreover, the prognostic value of identifying micrometastases and macrometastases should be quite different. However, current evidence is not strong enough to support this hypothesis^7^. Two reasons lead to this underappreciation of tumor-area-to-MLN-area ratio (T/MLN). One reason is that micrometastases are easily missed by pathologists, due to our visual system can easily miss small objects. Besides, precisely quantifying T/MLN is time-consuming, and thus significantly increases the workload of pathologists by ~3–5-fold. Digital pathological workflows offer significant potential for both avoiding misdiagnoses and accurately quantifying T/MLN in a timely manner.

Breakthroughs in digital image analysis and artificial intelligence (AI) have the potential to help pathologists accurately calculate T/MLN and simplify these time-consuming tasks. With the increasingly high capacity of whole-slide image (WSI) scanners^8^, a digital workflow for accurate gastric cancer staging is increasingly available. Deep learning has been successfully used for detection of LN metastases in women with breast cancer. The algorithm performance showed diagnostic accuracy comparable to pathologists^9^. Algorithm-assisted pathologists demonstrate higher accuracy than either the algorithm or the pathologist alone^10,11^.

Here, we show a deep-learning framework for analyzing LN WSIs of GC and calculating T/MLN to reduce the workload for pathologists and improve in TNM staging, ultimately bring about more precise therapeutic strategies for oncologists.

## Results

### Workflow for the automatic analysis of LN WSIs

**Fig. 1:**
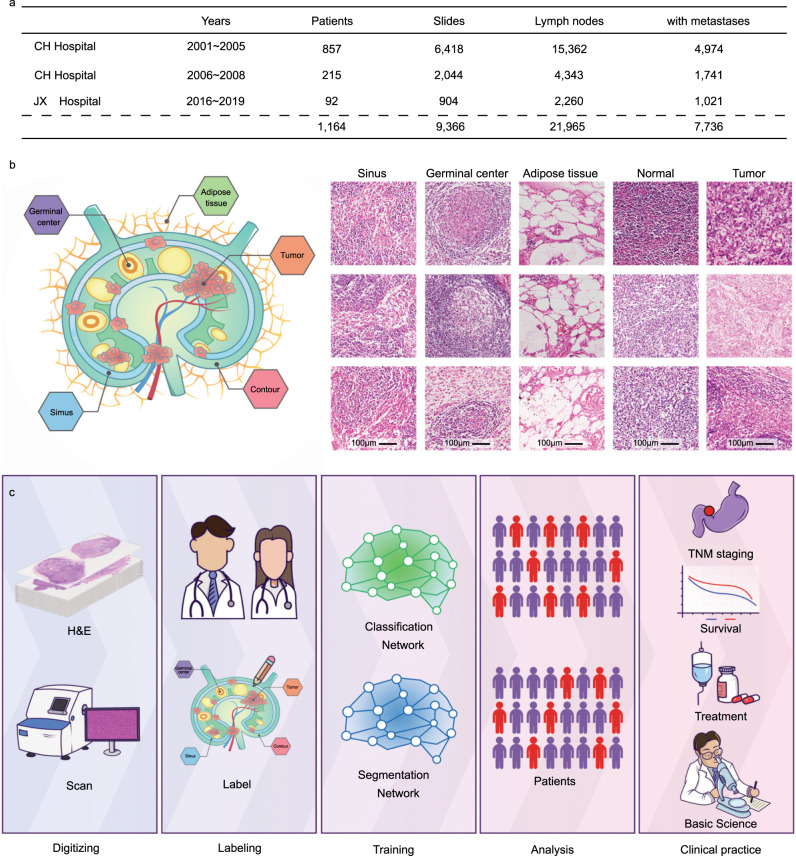
Data and workflow for analysis of cancer lymph node metastasis with deep learning. **a** Summary of each dataset. **b** Representative images for each of the five tissue classes we labeled in the lymph node area. **c** H&E pathological slides were first scanned to obtain WSIs. The WSIs were then labeled for training networks. The trained networks were used to analyze the patient’s WSIs and applied to clinical practice.

We focused on the Changhai (CH) Hospital 2001–2005 GC cohort for training and validation of the deep-learning framework, and the other two cohorts for testing the framework (Fig. 1a). In addition, we used the CH Hospital 2001–2005 GC cohort as the discovery cohort, and the CH Hospital 2006–2008 GC group and Jiangxi Provincial Cancer (JX) Hospital 2016–2019 GC group as the validation cohorts for prognostic analysis. Specifically, based on the dataset from CH Hospital 2001–2005, we selected 120 WSIs with tumor metastasis and 60 WSIs without tumor metastasis each year for training and validation to improve our framework robustness and avoid bias. The remaining slides were used as a test set. At the same time, from their clinical experience, doctors not only labeled the tumor area but also labeled the tissues that were easily misidentified by algorithms such as germinal centers and sinuses. As shown in Fig. 1b, the outer edge of each LN was labeled red, germinal centers were labeled blue, and tumor compartments were labeled yellow.

The workflow for our study is outlined in Fig.1c. First, we digitized H&E-stained LN pathology slides. Then, we selected a small number of samples for detailed annotation and trained the segmentation network and classification network (see “Methods” for details). With trained networks, we analyzed all WSIs. Next, we calculated the T/MLN for each GC patient based on the output of our system. Finally, based on the T/MLN, the overall survival of GC patients was analyzed by Kaplan–Meier (KM) analysis according to the N stage for each patient. In addition, we validated these results on two independent datasets: GC LNs from CH Hospital 2006–2008 cohort and JX Hospital 2016–2019 cohort.

### Deep-learning framework diagnosis of LNs with metastases

**Fig. 2:**
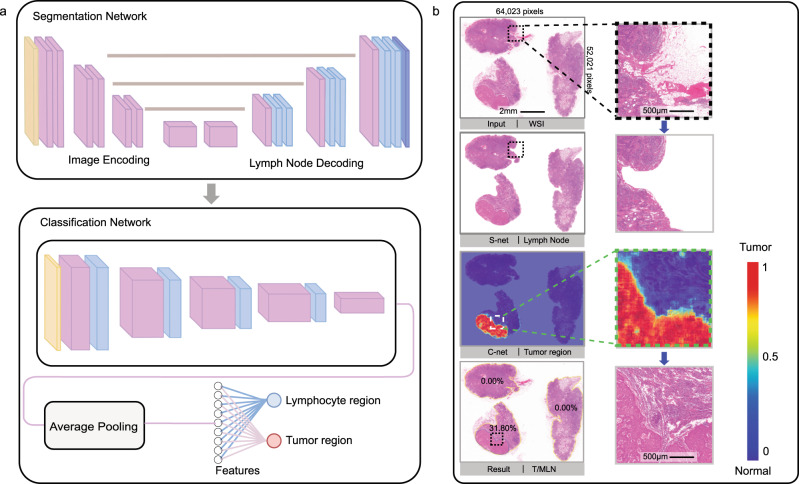
Deep-learning framework. **a** Slide analysis workflow. **b** Representative slide identified by deep learning. The slide is first input into the segmentation network to extract the lymph node region and remove tissues such as fat and muscle outside the lymph node. The tissues in the lymph node region are then classified by the classification network to identify the tumor region. The area ratio of tumor metastatic lymph nodes (T/MLNs) is finally calculated based on the heatmaps.

The deep-learning framework we developed is shown in Fig. 2. This framework consists of three phases—segmentation, classification, T/MLN calculation (see “Methods” for details). The LN segmentation network used the U-Net architecture to extract the LN regions from the WSIs 1× magnification thumbnails. The network was then fully trained through 700 marked WSIs including 1321 LNs. We tested the performance of the segmentation network on the validation set, and found a mean Jaccard index of 95.8%, and a mean Dice score of 98.6%. An example of the algorithm output is shown in Fig. 2b, where the adipose tissue and muscle fibers outside the LNs were excluded. After the AI-assisted diagnosis outputs the heatmaps, the pathologist reviewed the high-confidence area of the heatmap and corrects the wrong area on it.

As our classification network was used to analyze the WSIs of all LN tissues in each patient, the speed of inference and precision needed to be balanced. We conducted experiments with a variety of mainstream classification networks on an NVIDIA TITAN V GPU. These networks were fully trained with labeled WSIs. We tested the accuracy and inference speed of each network on the validation set, as shown in Supplementary Fig. 1. Compared to Inception V4^12^ and ResNet-101, ResNet-50 had a similar accuracy but a more efficient inference speed. Therefore, we used the Resnet-50 model as the classification network to analyze all the LN WSIs. We tested the performance of the classification network on the validation set and achieved a mean Dice score of 94.4%, a patch-level area under curve (AUC) score of 0.990, a slide-level AUC (nodal metastasis: present or absent) of 0.986, and an average free response-operating characteristic score of 0.872. An example of the output of the algorithm can be seen in Fig. 2b, which shows the degree of suspicion of the tumor area by heatmap.

**Fig. 3:**
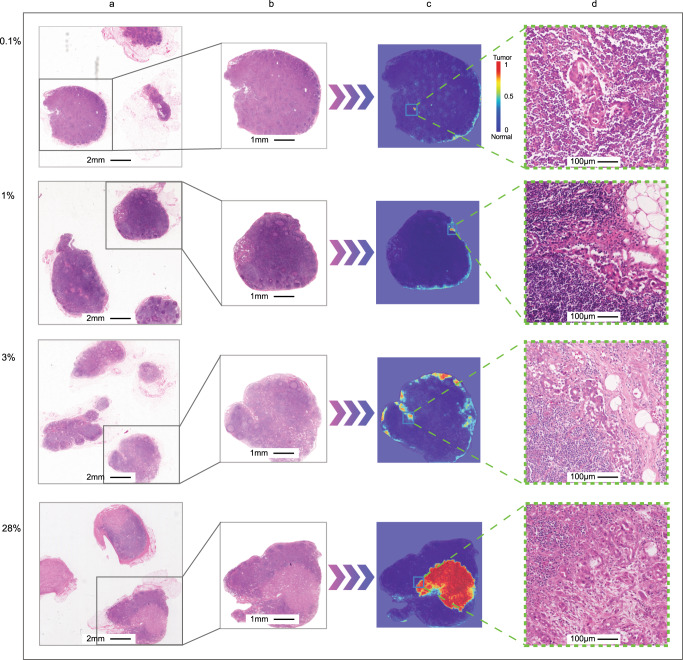
Visualization of the prediction results of four slides selected from the CH Hospital 2001–2005 cohort. We performed the analytical workflow on each slide to identify the lymph node areas of the gastric cancer and generate the heatmap of the tumor areas. We selected four slides with different tumor metastasis ratios. The redder the color, the higher the confidence of the tumor. **a** WSIs of lymph node tissue, **b** lymph node areas of segmentation network output, **c** heatmaps of classification network output, and **d** partial magnification of the detected tumor area.

After LNs outline and tumor composition were identified, it was straightforward for the computational analysis system to precisely calculate the proportion of tumor components and LNs (ranging from 0.01 to 100%). We observed that the first three rows in Fig. 3 are typical examples of micrometastases with a diameter of <2 mm, and the fourth row in Fig. 3 is a typical example of macrometastases with a diameter of >2 mm. Accurate calculation of T/MLN, especially for those < or ~2%, is beyond the ability of the human eye, while this is the strength of our algorithm.

### Improving diagnosis process with AI-assisted analysis

We then tested the performance of our framework with the original diagnosis by pathologists in the CH Hospital 2001–2005 GC cohorts. Because of loss, destruction, mildew, or thickened slides, 68 slides of 140 LNs in the archives were not available. Resliced or manually identified sections were used to avoid the potential biases caused by the above flaws to a degree. For the 64 slides of 128 LNs reserved above, we only diagnosed manually and detected 35 MLNs. In the end, 857 of 859 cases were available for further analysis. Supplementary Figure 2a, c shows that 94.5% (14,401/15,234) of LNs were consistent, and 86.8% (744/857) of cases’ N stage were consistent between the original diagnosis and the only AI diagnosis.

Pathologists have better specificity in the diagnosis of tumor tissues, while AI has better sensitivity and speed. The synergy of the combination of pathologists and AI is more clinically meaningful than the clinician versus AI comparison^10,13^. In addition, AI system does not need to completely surpass the level of the pathologist, which is also impossible, but to achieve the highest possible sensitivity with an acceptable false-positive rate^11^. In this study, two senior pathologists further reviewed all WSIs based on these heatmaps, which is the AI-assisted mode. Supplementary Figure 2b, d shows that while in 6.8% (360/5299) of the MLNs identified with AI assisted, the tumor lesions were not found by only pathologists, and in 1.5% (82/5299) of the MLNs diagnosed by pathologists, the tumor lesions were not found by only AI. In summary, the accuracy of only AI was 96.9% (14,761/15,234), sensitivity was 98.5% (5217/5299), and specificity was 96.1% (9544/9935). The sensitivity depended on the specific tumor type, and the missed diagnoses of AI were mainly mucinous adenocarcinoma and signet ring cell carcinoma (Supplementary Fig. 3).

This resulted in revised N-staging for 69 cases (8.1%) (Supplementary Fig. 2d). For the upstaging cases, the LNs that were incorrectly diagnosed by the pathologist came from missed diagnosis of micrometastasis (Supplementary Fig. 2e). The diagnosis of these micrometastases requires scanning the WSI at low and high magnification, which takes time and patience. This implies that our framework can help further study micrometastasis. For the downstaging cases, these were due to the loss of the original LNs or miscalculation of the amount of MLNs. Therefore, 4.6% (43/857) cases were under-staged by pathologists due to missed diagnosis of micrometastases, lower than the average level of published data (24%)^5^.

Actually, we observed how comparable the performance of the AI-assisted analysis was to manual estimation depended on the T/MLN (much better for AI assisted in <5% T/MLN, slightly better in 5–50% T/MLN, and no significant difference in ≥50% T/MLN).

Regarding time effort, it takes a pathologist 3–15 min to diagnose the N-staging of a single case, depending on the total number of resected LNs (ranging from 16 to 50) and the difficulty of classifying each LN. Furthermore, in order to calculate the proportion of MLNs, the pathologist also has to count all LNs and MLNs to estimate and record the proportion of each MLN, and then calculate the proportion of MLNs for each case. This is a time-consuming project for a pathologist. However, the processing time of one case with our framework depends on the number of LNs. Currently, the average consumption of computing classification probability for each batch patches of 128 was 430 ms on a single NVIDIA TITAN V GPU, and that for each case was about one minute. If the AI-assisted diagnostic mode is used, the review of macrometastasis takes almost no time, micrometastasis ~10 s, and each patient ~1 to 5 min. Therefore, only 2–6 min is needed for an AI-assisted pathologist to diagnose a patient’s LN WSIs.

The scanning time of LNs using KF-PRO-120 or NanoZoomer-S60 is currently 1–3 min for a slide of 3–5 LNs at ×20, and this high-throughput digital scanner can process ~2000 LNs from 600 patients per day. This represents the average demand at a tertiary medical center. Therefore, the scanning step is not the bottleneck for automatic computational analysis of N-staging.

### Predicting cancer prognosis with T/MLN

**Fig. 4:**
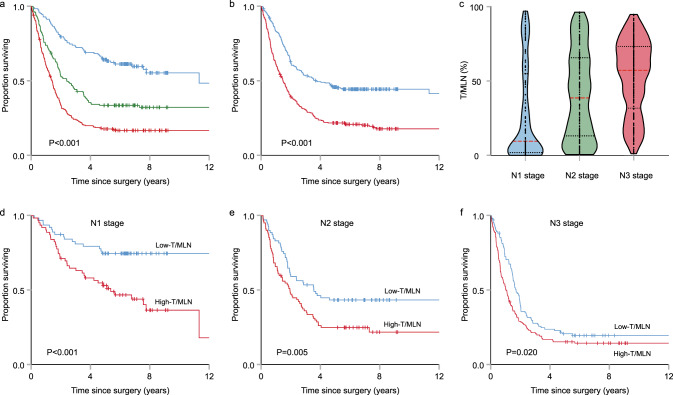
Kaplan–Meier analysis of cancer-specific survival and distribution statistics of T/MLN in the N stage with low-T/MLN and high-T/MLN at the CH Hospital 2001–2005 cohort. **a** KM curve with the N stage. **b** KM curve with the T/MLN. **c** Distribution of T/MLN with the N stage (*n* = 127 patients at N1 stage; *n* = 153 patients at N2 stage; *n* = 236 patients at N3 stage). In the violin plot, red lines indicate the median. **d** KM curve at N1 stage. **e** KM curve at N2 stage. **f** KM curve at N3 stage. *P* values were determined by two-sided log-rank test.

Accurate and efficient identification of MLN using our framework will greatly improve the work efficiency and reduce the rate of missed diagnosis by pathologists, thus possibly altering the workflow of pathologists. However, whether or not deep-learning based on LN analysis could be used for prognostic prediction remained to be demonstrated. The number of LNs and the ratio of MLNs, especially the former (Fig. 4a), are closely associated with patient outcomes in our study and other previous studies^6^. However, the current N-staging system ignores an important factor, the area of metastatic tumor cells in an MLN (T/MLN), which is difficult to acquire without using deep learning, but does correlate with cancer patient outcomes (Fig. 4b).

Based on the T/MLN from deep-learning precision calculations, we provide visualized evidence that with the improvement of N-staging, the T/MLN is likely to increase from 0.270 ± 0.318 at N1, to 0.395 ± 0.293 at N2, and to 0.517 ± 0.243 at N3. Moreover, even at the same stage, the T/MLN varies significantly from patient to patient (Fig. 4c). As demonstrated in Fig. 4c, half of the GC patients at the N1 stage had a T/MLN <5%, while the other half had a wide range of T/MLN values, ranging from 5 to 100%. We thus came to the hypothesis that there may be T/MLN-specific differences in the prognostic power; therefore, Cox regression analysis was performed using the median T/MLN (0.45) of the CH Hospital 2001–2005 cohort (Table 1). Using univariable analysis, we found that a higher T/MLN (>0.45) was correlated with poor outcome (hazard ratio [HR] = 2.05, 95% confidence interval [CI] 1.66–2.54, *P* < 0.001). To evaluate the independent prognostic ability of a T/MLN, we next performed multivariable analysis. In a multivariable Cox regression that included T/MLN, N stage, histological grade, age, size, histological type, Lauren type, pathological tumor stage, surgery type, blood transfusion, location. and sex, and T/MLN had an HR of 1.39 and a 95% CI of 1.10–1.75 (*P* = 0.007) (Table 1).

**Table 1:** Univariate and multivariate cancer-specific survival analysis of CH Hospital 2001–2005 gastric cancer cohort.

Variable	Univariable			Multivariable		
	HR	95% CI	*P* value	HR	95% CI	*P* value
T/mln (1: ≤0.45; 2: >0.45)	2.05	1.66, 2.54	<0.001	1.39	1.10, 1.75	0.007
N stage (1–3: n1–n3)	1.88	1.63, 2.16	<0.001	1.72	1.48, 2.00	<0.001
Pathological tumor (t) stage (1–4: t1–t4)	1.69	1.43, 1.99	<0.001	1.30	1.10, 1.576	0.004
Size (1: ≤5 cm; 2: >5 cm)	1.55	1.25, 1.91	<0.001	0.94	0.74, 1.18	0.577
Histological grade (1–3)	1.24	1.03, 1.49	0.023	1.30	1.07, 157	0.008
Surgery type (1: radical; 2: palliative care)	2.97	2.33, 3.77	<0.001	2.13	1.64, 2.76	<0.001
Age at surgery, years (1: ≤60; 2: >60)	1.66	1.34, 2.05	<0.001	1.33	1.07, 1.66	0.011
Sex (1: male; 2: female)	0.97	0.77, 1.21	0.767	–	–	–
Histological type (1: adenocarcinoma; 2: other)	1.01	0.76, 1.34	0.945	–	–	–
Lauren type (1: intestinal; 2: diffuse or mixed)	1.24	0.99, 1.55	0.061	–	–	–
Blood transfusion (1: no; 2: yes)	1.94	1.56, 2.41	<0.001	1.42	1.12, 1.80	0.004
Location_pylorus	–	–	0.003	–	–	0.161
Location_cardia	1.34	0.98, 1.84	0.065	1.04	0.76, 1.43	0.806
Location_whole stomach	1.82	1.29, 2.57	0.001	1.21	0.84, 1.32	0.313
Location_gastric body	1.03	0.79, 1.33	0.838	0.80	0.61, 1.04	0.096

*P* values were determined by two-sided log-rank test.

*T/MLN* ratio of tumor area to metastatic lymph node area, *HR* hazard ratio, *CI* confidence interval.

**Fig. 5:**
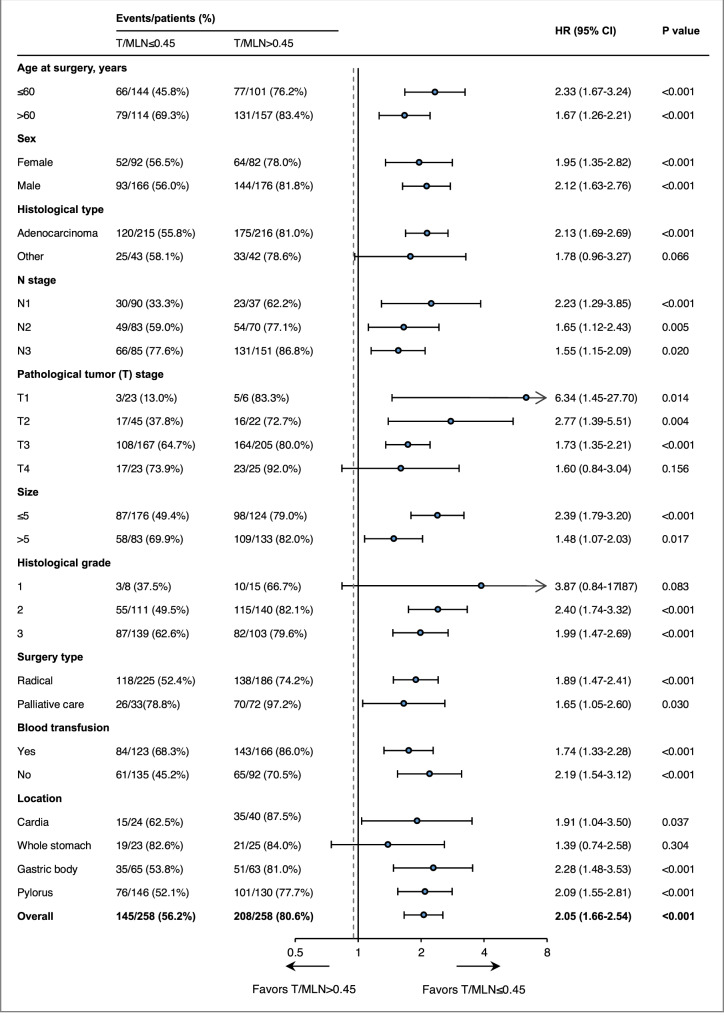
Forest plot of T/MLN for gastric cancer patients in the analysis of cancer-specific survival from the CH Hospital 2001–2005 cohort. HRs with 95% CIs in stratified survival analysis with higher T/MLN and lower T/MLN, including age, sex, histological type, N stage, pathological tumor stage, tumor size, histological grade, surgery type, blood transfusion, and location. *P* values were determined by two-sided log-rank test. Error bars represent the 95% CIs. HR hazard ratio.

In a stratified analysis of cancer-specific survival, the HR between gastric cancer patients with higher T/MLN and lower T/MLN was similar in a subgroup of each patient characteristic (Fig. 5). In a Cox regression model of N-stage grouping, cancer-specific survival of gastric cancer patients with higher T/MLN was shorter than that of gastric cancer patients with lower T/MLN (N1 stage: HR = 2.23, 95% CI 1.29–3.85, *P* < 0.001; N2 stage: HR = 1.65, 95% CI 1.12–2.43, *P* = 0.005; N3 stage: HR = 1.55, 95% CI 1.15–2.09, *P* = 0.020) (Fig. 4d–f). Thus, T/MLN can provide patients with more prognostic information based on N-staging.

Since the 7th AJCC TNM staging system^14,15^, the N3 stage is subgrouped to N3a (metastasis in 7–15 regional LN) and the N3b (metastasis in >15 regional LN). We found that the patients with low-T/MLN have a better prognosis than those with high-T/MLN in the N3a stages (HR = 1.47, 95% CI 1.06–2.04, *P* = 0.021) (Supplementary Fig. 4a). Due to the insufficient number of patients with N3b stage (46 patients), it is not significant in N3b stage (HR = 2.13, 95% CI 0.83–5.45, *P* = 0.108) (Supplementary Fig. 4b).

Whether micrometastases have the same prognostic value as macrometastases was not well-studied. Here, we analyzed the prognostic staging performance of LN micrometastasis with the records corrected by the “AI-assisted model.” As shown in Supplementary Fig. 4c, N-staging can be further grouped according to whether there is micrometastasis (*P* < 0.001), especially in the N1 stage. However, T/MLN is better than LN micrometastasis in improving the prognostic staging performance (*P* < 0.001) (Supplementary Fig. 4d). The *C*-index of only N stages is 0.646, N stages with micrometastasis are 0.654, and N stages with T/MLN are 0.694. This result indicates that the area, better than the diameter, of tumor cell clusters does reflect the degree of metastasis.

### Performance on independent cohorts

Our framework was then tested on independent datasets of LNs WSIs from the CH Hospital 2006–2008 GC cohort (215 cases, with 2044 WSIs and 4343 LNs) and the JX Hospital 2016–2019 GC cohort (92 cases, with 904 WSIs and 2260 LNs). As demonstrated in Supplementary Fig. 5, the performance of our framework remained satisfactory without transfer learning. For those WSIs from the CH Hospital 2006–2008 cohort (Supplementary Fig. 5a, b), the sensitivity of only AI was 97.9% (1730/1767) and specificity was 86.3% (2223/2576). For those from JX Hospital 2016–2019 cohort, the sensitivity of only AI was 96.0% (1013/1055) and specificity was 85.1% (1026/1205) (Supplementary Fig. 5c, d). The sensitivity of the framework was still high and the specificity was reduced, but it was within the acceptable range. We also randomly selected 100 slides from JX 2016–2019 cohort to be scanned on NanoZoomer-S60, and then analyzed after WSI standardization. The experimental results are shown in Supplementary Fig. 6a–e. We found that standardization can effectively alleviate the decrease in model performance caused by differences in scanning between different hospitals and different scanners. However, we also found that several factors affect the performance of the framework, including very poor staining of H&E-like dull staining, uneven staining, or air bubbles, and poorly differentiated tumors are significant obstacles to proper identification. For the better application of AI-assisted diagnosis in the clinic, a standard process for the preparation of H&E slides should be established and popularized in the pathological workflow.

We then validated the association between the T/MLN and prognosis of T/MLN in these two cohorts, and the results were similar to the CH Hospital 2001–2005 cohort (Supplementary Fig. 7 and Supplementary Table 2).

Our result is one of the very interesting findings to date based on. The algorithm solved a problem observed before that remained difficult to address, namely, the association between the number of metastatic tumor cells and prognosis. All of these findings demonstrate the importance of T/MLN in predicting the outcome of patients with gastric cancer, which will serve as a potential complement to the current AJCC TNM staging system.

### Predicting tumor metastasis from WSIs

**Fig. 6:**
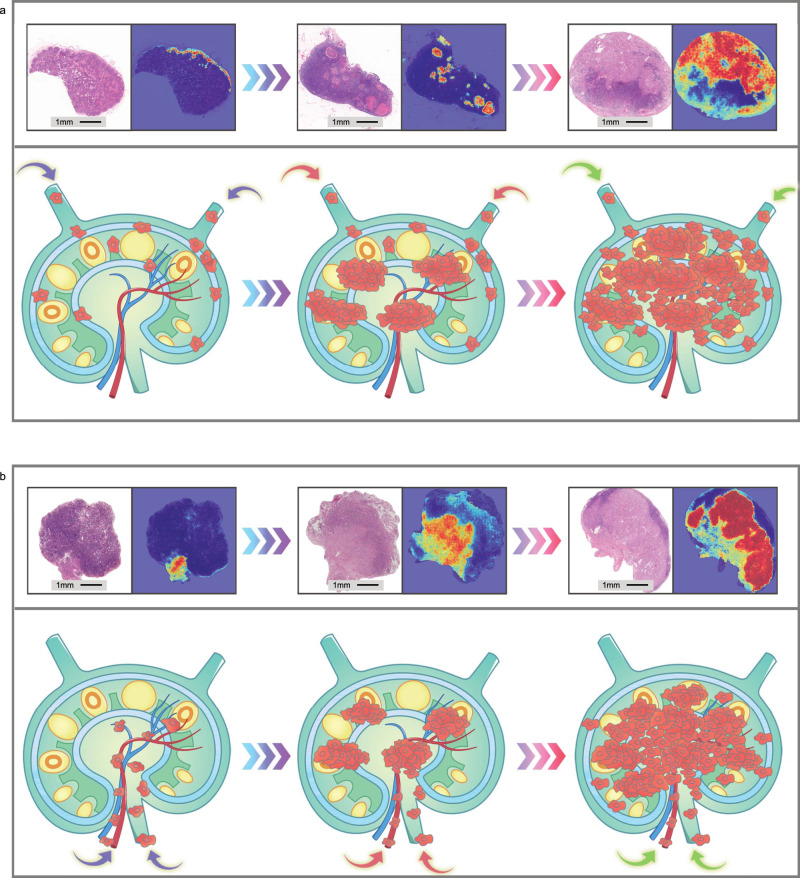
Visualization of spatial information of metastatic lymph node displaying the potential process of tumor cells spreading in lymph nodes. **a** Representative images of HE slides and heatmaps of MLNs and diagrammatic sketch of MLNs showing that tumor cells invaded lymph nodes through afferent lymphatic vessels and gradually eroded the whole lymph nodes. **b** Representative images and heatmaps of MLNs and diagrammatic sketch of MLNs showing that tumor cells invaded lymph nodes through the hilum of lymph nodes.

The visual prediction power of our system helps to demonstrate the multidimensional spatial information presented in sections of LNs with metastasis. On the resulting heatmaps, each patch has a color that is proportional to the probability of the tumor components. The distribution of different colors represents the visual trajectory of tumor cells invading the LN. Heatmaps of these LNs indicates that the majority of tumor cells eroded LNs beginning from afferent lymphatic vessels around the LN (Fig. 6a). The hilum of a LN is a passage of blood vessels, nerves, and efferent lymphatic vessels. As demonstrated in Fig. 6b, a number of tumor cells eroded the LN beginning from the hilum. After eroding a LN either from lymphatic vessels or from the hilum, the tumor cells gradually erode the internal tissue of a LN until all of the LN is metastatic. Previous studies have shown associations between spatial information of tumor-infiltrating lymphocytes (TILs) and specific patterns of breast cancer and colorectal carcinoma (hot, altered, and cold tumors), which were both prognostic and predictive^16^. This signature of TILs can be classified using deep-learning on pathology images^17^. More recently, a deep convolutional neural network (Inception V3) has been used to correlate genotype–phenotype information from non-small cell lung cancer cases based on WSIs^18^. Therefore, our system visualization tools are powerful in extracting hidden features from WSIs of H&E-stained cancer tissues.

We found that the frequency of T/MLN varied significantly (N1 stage: *P* = 0.015; N2 stage: *P* < 0.001; N3 stage: *P* < 0.001) between D stations (lesser curvature of stomach) and E stations (greater curvature of stomach) (Supplementary Fig. 8a). We performed a survival analysis based on the differences in T/MLN between D-station and E-station LNs in gastric cancer patients (Supplementary Fig. 8b–d). We identified that cancer-specific survival was shorter in patients with D-station T/MLN values less than the corresponding E-station value in N1 stage and N2 stage (N1 stage: HR = 1.46, 95% CI 0.82–2.59, *P* = 0.195; N2 stage: HR = 1.23, 95% CI 0.82–1.86, *P* = 0.313), whereas patients in N3 stage showed the opposite trend (HR = 0.85, 95% CI 0.63–1.13, *P* = 0.260). Although not significant, considering that HRs is less informative in the case of the survival curves crossed at certain time points, we did find a potential tendency with the survival curves.

## Discussion

Our study demonstrates that deep-learning framework is useful for detecting LN metastases of gastric cancer from whole histopathology slides. The high performance (sensitivity 98.5%, specificity 96.1%) of our framework not only helps resolve the time-consuming workflow of identifying metastasized LNs from a large amount of resected LNs and calculating the total number of metastasized LNs, but also provides an objective and reproducible way to assess the proportion of tumor cells in each LN. Interestingly, ~6.8% of the LN WSIs misdiagnosed by a pathologist were corrected by our framework, suggesting that our framework would be helpful in assisting pathologists in their diagnoses. The misdiagnosis matrices in Supplementary Fig. 2 detail the discrepancies between original diagnosis and the output of our framework followed by a pathologist’s confirmation, and shows several representative examples in which our framework correctly detected tumor cells missed by the initial diagnosing pathologist. Without any doubt, these slides show micrometastases with very few and poorly differentiated tumor cells, which also scatter in the slide without gland formation—a classic histological feature of adenocarcinoma.

The widely accepted AJCC N-staging classification of the stomach is based on the number of LN with metastases, which requires the status of each LN and the total number of LNs recovered to be known for the selection of the appropriate N stage. Generally, a minimum of 15 resected LNs is recommended for adequate staging, which also contributes to superior overall survival after gastrostomy. N classifications based on the number of metastasized LNs neglect an important detail, T/MLN. Before actions are taken in a clinical practice, the knowledge of the precise quantity of T/MLN should resolve two questions. The first is to develop an objective and reproducible way to access the proportion of MLNs, which has been resolved by our deep-learning-based method. The second is to confirm the therapeutic and prognostic value of T/MLN. The diversity and heterogeneity of tumor tissues hides significant prognostic information, including stroma–tumor ratio, necrosis, cancer embolus, neural invasion, blood vessels, and inflammation, many of which have been only identified via deep learning. In various tumors, the stromal compartment and TILs can be quantified and visualized from H&E images using deep-learning models that can also predict patient outcomes and/or response to immunotherapies^17,19^. In addition, there are some works that directly extract features from the original pathological image for prognosis prediction^20–23^. These works have indeed proved that some features of pathological images are related to the prognosis. However, they have a common problem is that the interpretability is very weak, which makes them clinically unavailable. In the present study, we accurately calculated the proportion of tumor cells in each MLN and validated our deep-learning-based assessment as a prognostic marker for human GC. In a stratified analysis of cancer-specific survival, the HR between gastric cancer patients with higher T/MLN and lower T/MLN was similar in subgroups of each patient characteristic. To avoid bias based on the number of LNs, we further performed survival analysis using T/MLN on the basis of N stages. Not surprisingly, we found that deep-learning-determined T/MLN values are highly capable of predicting outcomes for GC patients at each N stage, especially at the N1 and N2 stages. In addition, we also confirmed that micrometastasis can indeed improve prognosis prediction. However, it is impossible to calculate the diameter of disseminated metastases manually (Supplementary Fig. 10a). For tumor cells that metastasized along the subcapsular sinus, although long in diameter, the areas are small, which could not reflect the real status of LN metastasis (Supplementary Fig. 10b). Moreover, T/MLN has better prognostic performance than micrometastasis in subdividing patients at the same stage into two groups with different outcome. Therefore, our two research questions were answered, and our deep-learning-based algorithm could precisely give an objective pathological evaluation of T/MLN, with T/MLN being prognostic of OS in GC patients. Another two independent GC cohorts during a later period yielded similar results with our algorithm. Therefore, although it is necessary to validate its prognostic value in larger and more diverse cohorts from other hospitals, we suggest that T/MLN values may be of great utility when incorporated into existing clinical N-staging workflows because of their convenience, reliability, and strong prognostic power.

Another advantage of our deep-learning framework is its ability to identify hidden information from medical imaging in human solid tumors. In the present study, visualized MLN demonstrated two potential patterns for the metastatic modes of tumor cells eroding LNs. This information will be of great help in investigating the underlying mechanisms of tumor metastasis. Overall, our study confirmed that a deep-learning framework is a useful tool for assisting pathologists and oncologists in their diagnosis and evaluation of WSIs of LNs, accompanied by providing quantitative and spatial assessments in the associated heatmaps. This information can be crucial in selecting appropriate therapeutic strategies and predicting the overall survival of GC patients.

The main limitation of this study is that our prognostic analysis of T/MLN was a retrospective dual-center retrospective study of gastric cancer from one nation. Whether it impacts the generalizability of our algorithms in other regions and whether it affects subsequent treatment also requires large-scale clinical trials. In the future, we will validate this algorithm in large and separate cohorts of various cancer patients from various regions. We will also compare whether the sensitivity of our AI assistant system is equivalent to immunohistochemical (IHC) staining. Moreover, we will extend this recognition to histological subtypes and Lauren classification of GC, as well as to non-neoplastic characteristics, including necrosis, fibrosis, and TILs in the tumor microenvironment. Recently, the International Gastric Cancer Association proposed a new GC staging system. This system shares the same TNM classification with the AJCC7 system, but introduces N3a and N3b into the staging^24^. Some clinical studies have also confirmed that this system improves the prognostic prediction performance of TNM classification^25^. We believe that T/MLN can be introduced into the TNM classification after conducting a prospective multicenter clinical trial.

We believe that T/MLN is only one of these indicators, and there will be more quantitative indicators to improve prognosis staging. Finally, we will establish a cloud-based platform where the WSIs of LNs will be passed to this platform with permission, and our algorithm will automatically recognize MLN and give accurate T/MLN. With the increasing amount of data available in the future, we hope that this computational approach will help pathologists and clinicians develop more accurate sub-N-staging, thereby improving treatment decisions and outcomes for patients.

## Methods

### Dataset

WSIs of LNs of GC were obtained from CH Hospital and JX Cancer Hospital. Characteristics of dataset and the overall computational strategy are summarized in Fig.1 and Supplementary Table 1. We only included patients with a malignant tumor of epithelial origin. Patients treated with neoadjuvant therapy were excluded. Images of H&E-stained, formalin-fixed, paraffin-embedded sections of diagnostic LNs from these cohorts were reviewed to choose images without tissue processing artifacts (bubbles, section folds, and poor staining). Since the importance of each single LN in N-staging, these slides with severe artifacts were resliced and restained. Two pathologists performed an initial quality review of 2024 cases. Only these with a total number of resected LNs over 7 and good quality were enrolled in this study. Finally, a total of 21,965 LNs from 1164 patients’ 9366 slides were selected, out of which, 7736 had metastatic lesions. According to the 8th AJCC TNM staging system^26,27^, we revised the original N-staging for all patients. The GC LNs used comprised of three cohorts: one from CH Hospital during 2001–2005 (15,362), one from CH Hospital during 2006–2008 (4343) and one from JX Hospital during 2016–2019 (2260) (Fig.1a). These LN slides were digitalized according to standard protocols to obtain WSIs^28^. Among these cases, we selected GC cases with follow-up records and at least one tumor metastatic lesion for prognostic studies (Supplementary Table 1). Detailed information of the GC cases from CH Hospital 2001–2005 had been published previously^29^, and re-followed up over the period from 2010 to 2012 in 516 of these cases.

### Data preparation

#### Data annotation

The dataset for training networks was manually annotated by pathologists using a web-based annotation program we developed. We train pathologists to annotate by pen on the iPad. Next, we developed the labeling protocol: Each WSI was annotated in detail by a pathologist. The pathologists annotate the four types of tissue with different colors, and finally annotate the outline of the LNs (Fig. 1b). For tumor tissue, the pathologist needs to label all. For easily misidentified by algorithms such as germinal centers, fat, and sinuses, pathologists should also label as much as possible. For these difficult to judge tissues, the two pathologists would discuss it to give a final result.

#### Data standardization

The staining of digital tissue slides is filled with undesirable color changes due to differences in raw materials, staining protocol, digital scanners, and fading from long-term storage. In order to analyze WSIs from different sources using our framework, we first standardized the staining of WSIs using SPCN^30^ based on CH 2005 WSIs. Due to the WSI background digitized by the scanner is not really white, we set the RGB channel cutoff of the background color to [210, 210, 210] according to the statistical results. When calculating the global stain color appearance matrix *W*, we sampled 32 patches with the background pixel ratio <0.2 in each WSI. At the same time, when calculating the global stain density map matrix *H*, we chose the robust pseudo-maximum of each row vector at 99.9%. Our data standardization was performed at ×20 magnification, which shortens the processing time by nearly five times, compared to ×40 magnification (original image).

In addition, there are differences in the specimen-level pixel size of different scanners. The ×40 objective lens of the Jiangfeng scanner is 0.2513 μm × 0.2513 μm, and the Hamamatsu scanner is 0.2206 μm × 0.2206 μm. We also standardized the WSIs of the Hamamatsu NanoZoomer-S60 scanner based on the Konfoong KF-PRO-120 scanner.

### Training segmentation network

The dataset we used to train the LN segmentation network included 900 WSIs with LN markers. We randomly selected 700 WSIs for training and 200 WSIs for validation. We used Openslide to extract ×1 magnification thumbnails and generate a mask based on the doctor’s markup for each thumbnail. In addition, to avoid the influence of visible variabilities in staining on our model, we converted the data into grayscale in the data preprocessing stage using decolorization^31^.

We adopted the U-Net architecture for the segmentation network, which included an encoding module, a decoding module, and shortcut connections between blocks of the same level and different paths. The encoding and decoding modules extracted semantic information layer by layer so that the model extracted rich features, and the skip connection combined low-level semantic features and high-level semantic features to make the model more sensitive to texture and other information. During the training process, we cropped images to 700 × 700 pixels, used random cropping and rotation to augment our dataset. We also used a cross-entropy loss function to calculate loss and used a stochastic gradient descent with a momentum of 0.9, a weight decay of 1e − 4, and a batch size of 32 during training. The initial learning rate was 0.001, and was then set to 0.001/2 after 20% of total iterations, 0.001/8 after 40%, 0.001/16 after 60%, and 0.001/32 after 80%. The training process was iterated 12,000 iterations.

### Training classification network

Our dataset for training classification network included 900 labeled WSIs, including 300 WSIs without tumor metastases and 600 WSIs with tumor metastases. We randomly selected 500 WSIs with tumor metastases and 200 WSIs without tumor metastases for network training, and the remaining 200 WSIs were used to verify the network performance. The background area (non-LN area) of each WSI was excluded based on the LN mask. We cut these WSIs in 768 × 768 pixels windows at a magnification of ×20 with sliding step of 768 using the Openslide. Each 768 × 768 patch consists of nine 256 × 256 patches.

We implemented the classification network to classify LN regions into tumor regions and lymphocyte regions. During training, we used color jitter in torchvision transforms with parameters: brightness with a maximum delta of 64/255, contrast with a maximum delta of 0.75, saturation with a maximum delta of 0.25, and hue with a maximum delta of 0.04. Patches were also randomly flipped and rotated with multiplies of 90°.

Our experiments used binary labels, and a patch was called positive if the center point was annotated as tumor. Due to the imbalance between the number of positive and negative samples, we sampled the negative samples in each epoch to ensure a positive and negative sample balance during training.

In order to improve the learning effect of the network on unconventional lymphocytes, such as germinal centers and sinus tissues, which are easily misidentified, we first added all these tissues to the negative sample set, and then sampled from other normal tissues during each epoch. We used the neural conditional random field^32^ as the classification network. VGG19^33^, AlexNet^34^, ResNet-18^10^, ResNet-34^10^, ResNet-50, ResNet-101^10^, Inception V3^35^, Inception V4, and MobileNet V2^2^ was used to extract features of patches, and the conditional random field was used to model the spatial correlation of patches. The output of the last layer of the network was the confidence of the tumor regions. The parameters of each network were initialized using the ImageNet dataset pre-trained model. We calculated the loss using the cross-entropy between the predicted probability and the real label, and used a stochastic gradient descent with a momentum of 0.9, a weight decay of 1e − 4, and a batch size of 1024 during training. For each network, the initial learning rate was 0.001 and was then set to 0.001/2 after 10% of the total iterations, 0.001/4 after 20%, 0.001/8 after 40%, 0.001/16 after 60%, 0.001/32 after 80%, and 0.001/64 after 90%. The training process was iterated 80,000 iterations.

We then calculated the classification performance and reasoning efficiency of each network, and finally selected ResNet-50 as the feature extraction module of the classification network for subsequent experiments.

### Performance verification

#### Reference standard

Two senior pathologists reviewed all slides to generate the reference standard for these datasets. Due to high specificity of senior pathologists in diagnosing tumor metastasis within LNs, most slides can be precisely diagnosed based only on H&E. In clinical practice, IHC staining is considered to be the most accurate method for assessing metastasis^9,36,37^. In our study, we used IHC (*CAM5.2, MAB-0687*, Fuzhou Maixin Biotech. Co., Ltd) restaining to resolve the slides of diagnostic uncertainty, without using for obvious metastases (Supplementary Fig. 9). Finally, we restained a total of 50 WSIs.

#### Only AI mode

We analyzed 9366 WSIs of LN tissue in total. We first resized these WSIs to a magnification of ×1 using the Openslide library, and then input these to the segmentation network to get LN segmentation masks. Next, we cut each WSI sequentially with a sliding window of 768 × 768 pixels with a sliding step of 256 at ×20 magnification and excluded patches outside the valid area of the LN segmentation mask. We input the patches of each WSI into the classification network and then obtained the classification confidence of each patch. We stitched the classification confidences together based on the position of each patch and obtained a heatmap for each WSI.

Since it is difficult for us to thoroughly annotate all WSIs, we used the MLN in each WSI from the corrected clinical record as labels and calculated the MLN of each WSI as the predicted value to test the performance of only AI.

#### AI-assisted mode

Three senior pathologists were involved in this study. Based on the heatmap output by the framework, two of them who also made the reference standards before this reviewed the original WSI region. First, the pathologists checked the area of the original WSI highlighted in the heatmap to determine whether it was tumor tissue, and then quickly reviewed the suspicious tumor area with reference to the heatmap. Finally, the pathologists referred to the reference standard to confirm the existence of missed diagnosis, and manually corrected the wrong area of the heatmap. The third pathologist was required to help identify the result in which the AI and pathologist’s cognition were inconsistent. If in doubt, we used IHC to restain to give the final result. We define the above process as the AI-assisted mode.

### Obtaining T/MLN for all patients

We used the trained networks to analyze all of the LN WSIs from the CH Hospital 2001–2005 GC cohort, the CH Hospital 2006–2008 GC cohort, and the JX 2016–2019 GC cohort.

The T/MLN of LN level is a ratio of the area of the tumor regions of a MLN to the area of that MLN. T/MLN of patient level is the average of T/MLN for all of the MLNs from each patient.

Based on the heatmaps pathologists reviewed, we calculated the area of the LN and tumor (0.5 as the classification threshold) for all of the MLNs from each patient. We then figured out the T/MLN using the Eq. (1),

1 $${\mathrm{T/MLN}} = \frac{1}{m}\mathop {\sum }\limits_{i{\mathrm{ = 1}}}^m \left( {\frac{{A_{{\mathrm{tumor}}}^i}}{{A_{{\mathrm{MLN}}}^i}}} \right)$$

where *m* is the number of MLNs, *A*_tumor_ is the total number of tumor pixels in each MLN, and *A*_MLN_ is the total number of pixels of that MLN.

### Statistics and reproducibility

At the end of the training phase, we used the validation set to evaluate the performance of our segmentation and classification networks. The validation set contained 200 WSIs. For the segmentation network, we generated 200 ×1 magnification thumbnails as inputs. We used the Dice score and Jaccard index^38^ to evaluate the performance of segmentation network with 0.5 as the segmentation threshold. For the classification network, we used the Dice score and the metrics employed in CAMELYON16 challenge to evaluate our classification model^8^. For slide level, the AUC score, accuracy, sensitivity, and specificity (0.5 as the classification threshold) was used for performance evaluation. For patch level, the average free response-operating characteristic curve was used for evaluation, which was defined as the average detection sensitivity at six predefined false-positive rates per slide: 1/4, 1/2, 1, 2, 4, and 8. At the same time, for patch level, we also evaluated AUC, which is important for accurate calculation of T/MLN.

We then measured the cancer-specific survival for each patient, as it is considered to be the most common clinically relevant endpoint for GC patient cohorts. Here, the definition of an event was limited to the death of the same cancer patient. Follow-up time was calculated from enrollment to death or loss of follow-up. When HRs are less informative in the case of the survival curves crossed at certain time points, the survival curves will provide more intuitive information^39^. Clinical and pathological markers were included in the multivariate analysis. We also used Harrel’s concordance index (C-index) as a metric for assessing the predictive performance. The two-sided Mann–Whitney test, Wilcoxon’s matched-pairs signed-rank test, and Mantel–Cox log-rank test were used as appropriate. A two-sided *P* value < 0.05 was considered statistically significant.

We tested the performance of the deep-learning framework and the prognostic predictions using three independent cohorts without retraining the networks. All attempts at replication were successful with similar results.

### Hardware and software

The segmentation network and classification network were trained using PyTorch v1.3^40^ on servers equipped with eight NVIDIA TITAN V GPU cards. All slides were digitized using KF-PRO-120, and part of the slides was also scanned using NanoZoomer-S60. WSIs were formatted using OpenSlide (https://openslide.org/). SPSS 25.0 was used for survival analysis. Scikit-learn was used to calculate the AUC.

### Ethical compliance

All patients in this study signed an informed written consent form before the operation, which contained a statement on the pathological tissue and clinical data for clinical research. This study was approved by the ethics committees of the Changhai Hospital and Jiangxi Provincial Cancer Hospital.

### Reporting summary

Further information on research design is available in the Nature Research Reporting Summary linked to this article.

## Supplementary information

Supplementary Information

Reporting Summary

## Data Availability

The publicly shared gastric cancer metastases imaging dataset to test the models in this study is available at 10.6084/m9.figshare.13065986. The dataset consists of 500 WSIs of lymph node specimens, including 250 with tumor metastasis and 250 without tumor metastasis. The dataset is accessed under the approval of the Ministry of Science and Technology of China (authorization number, 2020BAT1012). The remaining datasets are not publicly available due to hospital regulations and patient privacy. Source data are provided with this paper. The remaining data are available within the Article, Supplementary information, or available from the authors upon request.

## Source data

Source Data

## Code availability

All code related to this method was written in Python. Custom code related to the deep learning models is available at https://github.com/MHMAILab/auto_lymph.
