# Supplementary material for: Predicting gastric cancer outcome from resected lymph node histopathology images using deep learning
Source: Nat Commun. 2021 Mar 12;12:1637. doi: 10.1038/s41467-021-21674-7 (PMC7954798; doi:10.1038/s41467-021-21674-7)
Supplement: Supplementary file 1 — Supplementary Information [file 41467_2021_21674_MOESM1_ESM.pdf]

Supplementary Information

a

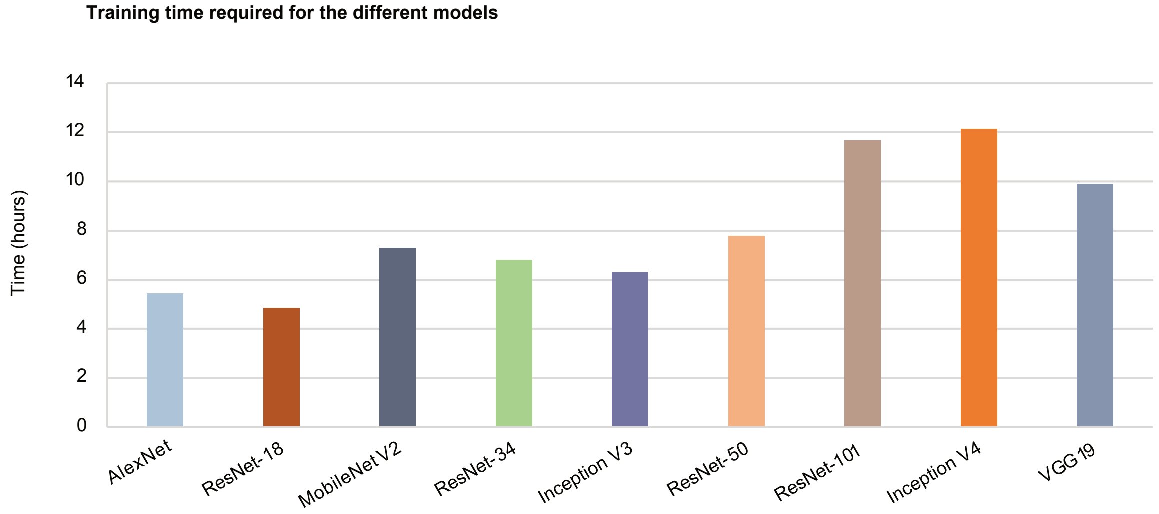

b

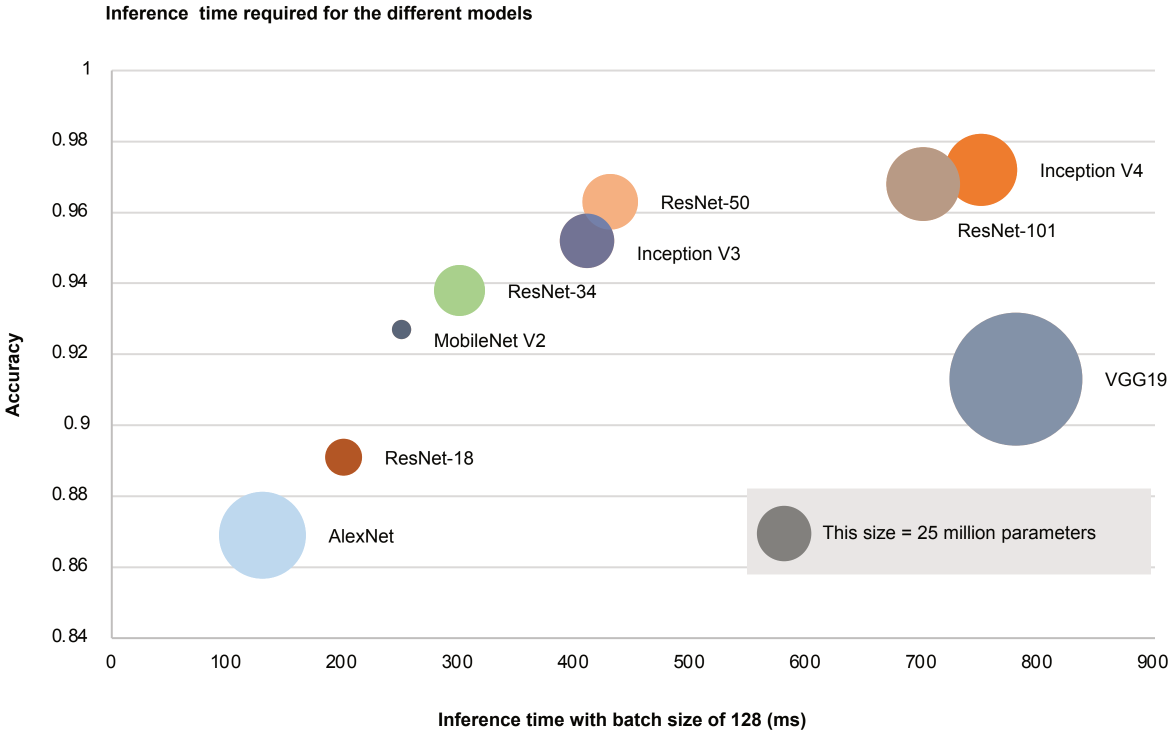

Supplementary Fig. 1. **Comparison of nine deep convolutional neural networks.** We experiment on nine mainstream convolutional neural networks (VGG19, AlexNet, ResNet-18, ResNet-34, ResNet-50, ResNet-101, Inception V3, Inception V4, MobileNet V2). (a) Comparison of the training time. (b) Comparison of the accuracy and inference efficiency. We compared the accuracy and inference efficiency of the nine mainstream convolutional neural networks on the data sets of gastric cancer lymph nodes. We experimented on a Nvidia Titan V GPU with batch size of 128. At the same time, we calculated the number of parameters for each network. The size of the circle in the figure represents the amount of the parameter. The accuracy of ResNet-50, ResNet-100, and Inception V4 is more than 0.96, but the inference time of ResNet-50 is half that of the other two networks.

a

		Only AI Diagnosis	
		0	1
Original Diagnosis	0	9,544	751
	1	82	4,857

b

		AI-assisted Diagnosis	
		0	1
Original Diagnosis	0	9,935	360
	1	0	4,939

c

N-stage	Original diagnosis	Only AI Diagnosis			
		N0	N1	N2	N3
N0	202	163	31	6	2
N1	149	2	124	17	6
N2	195	0	5	170	20
N3	311	0	1	6	304

d

N-stage	Original diagnosis	AI-assisted Diagnosis			
		N0	N1	N2	N3
N0	202	192	9	1	0
N1	149	0	135	14	0
N2	195	0	2	177	16
N3	311	0	0	5	306

e

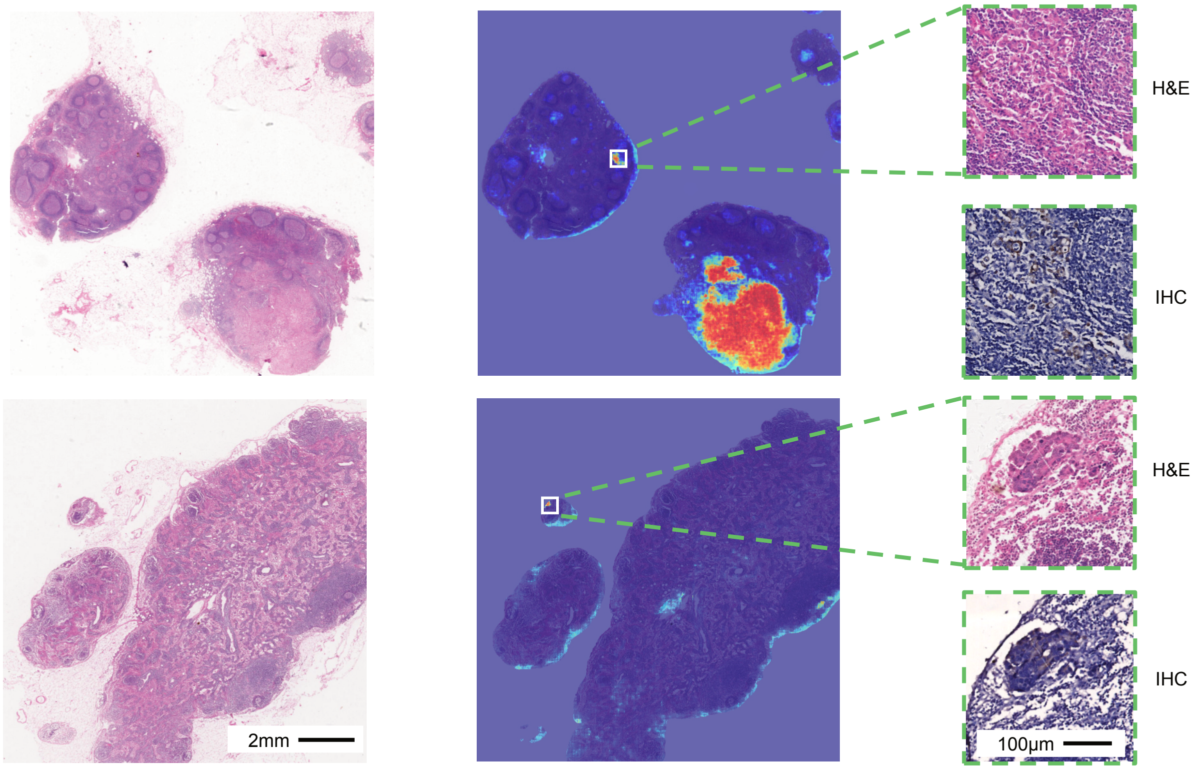

Supplementary Fig. 2. **Results of deep-learning framework performance analysis and visualization of same cases of the gastric cancer lymph nodes metastasis missed by clinicians from the CH Hospital 2001~2005 GC cohort**. (a) Comparison MLN of only AI diagnosis with original diagnosis of the pathologist (n=15,234 lymph nodes). (b) Comparison MLN of AI-assisted diagnosis with original diagnosis of the pathologist. (c) Comparison N-stage of only AI diagnosis with original diagnosis of the pathologist (n= 857 patients). (d) Comparison N-stage of AI-assisted diagnosis with original diagnosis of the pathologist. (e) The tumor metastasis in the lymph node region where the white box of the middle heatmap is missed by the clinician but our framework detected it. We confirmed by immunohistochemical (IHC) restaining.

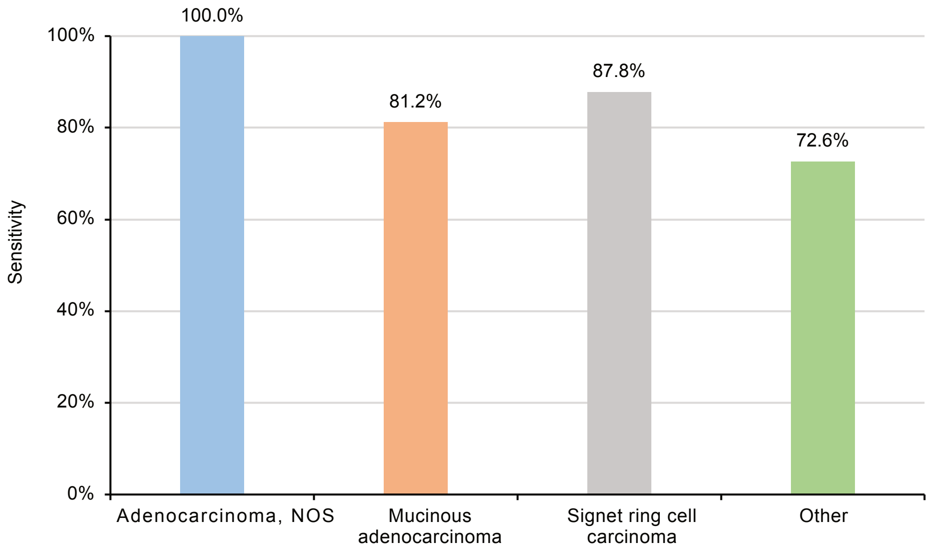

Supplementary Fig. 3. **AI-system's sensitivity in different types of tumor detection from the CH Hospital 2001~2005 GC cohort.** We performed statistics on the model performance of different types of gastric cancer tumors (n=4,667 MLNs of adenocarcinoma, NOS; n=431 MLNs of mucinous adenocarcinoma; n=139 MLNs of signet ring cell carcinoma; n=62 MLNs of other types).

NOS, not otherwise specified.

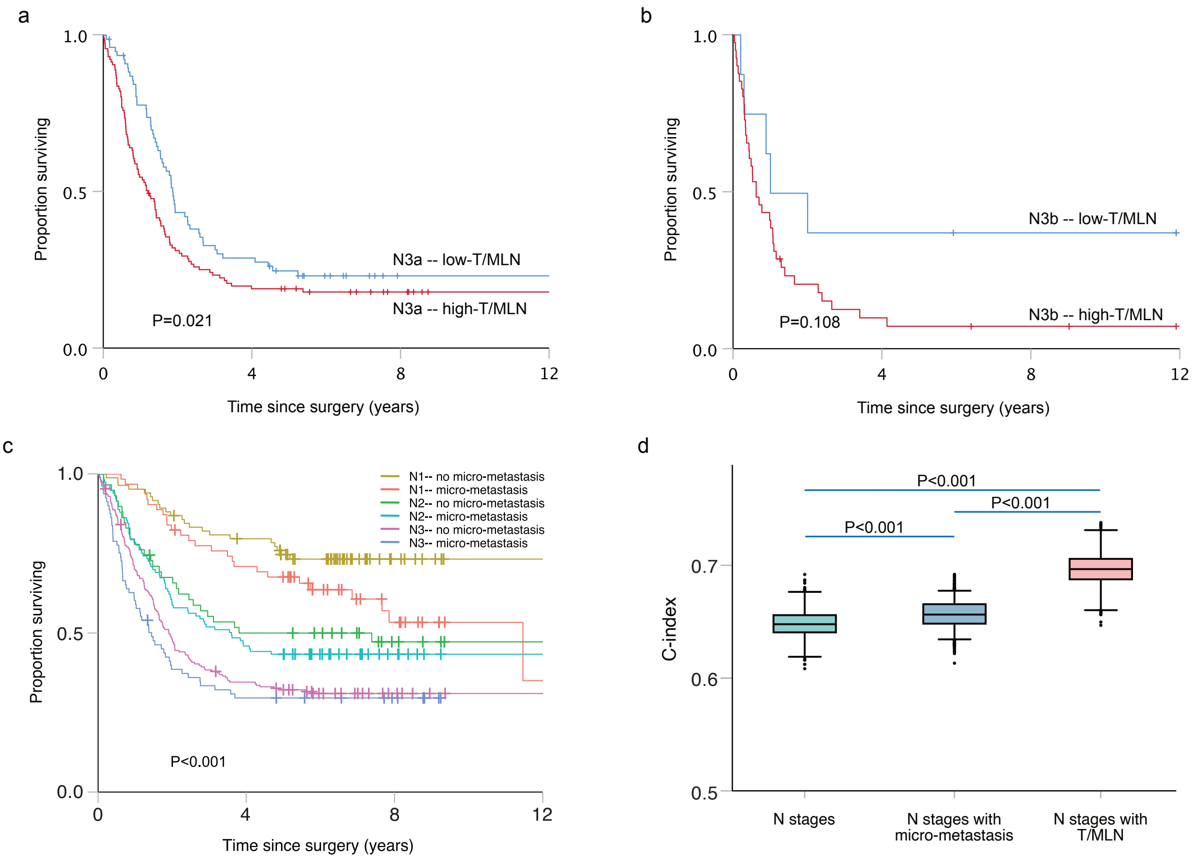

Supplementary Fig. 4. **Cancer-specific survival analysis in N3 stage with low-T/MLN and high-T/MLN and in the N-stage with lymph node micro-metastasis from the CH 2001~2005 GC cohort**. (a) KM curve in the N3a stage. (b) KM curve in the N3b stage. (c) KM curve with lymph node micro-metastasis in the N-stage. (d) Comparison of the performance of micro-metastasis and T/MLN in predicting prognosis through C-index in the N-stage (n=516 patients). For the boxplots, whiskers represent the ranges. The middle line within the box represents the median. The upper and lower boundaries of the whiskers represent the 5th and 95th percentiles, respectively. *P* values were determined by two-sided log-rank test (a-c) or two-sided Mann-Whitney test (d).

a

		Only AI Diagnosis	
		0	1
Original Diagnosis	0	2,223	379
	1	37	1,704

b

		AI-assisted Diagnosis	
		0	1
Original Diagnosis	0	2,576	26
	1	0	1,741

c

		Only AI Diagnosis	
		0	1
Original Diagnosis	0	1,026	213
	1	42	979

d

		AI-assisted Diagnosis	
		0	1
Original Diagnosis	0	1,205	34
	1	0	1,021

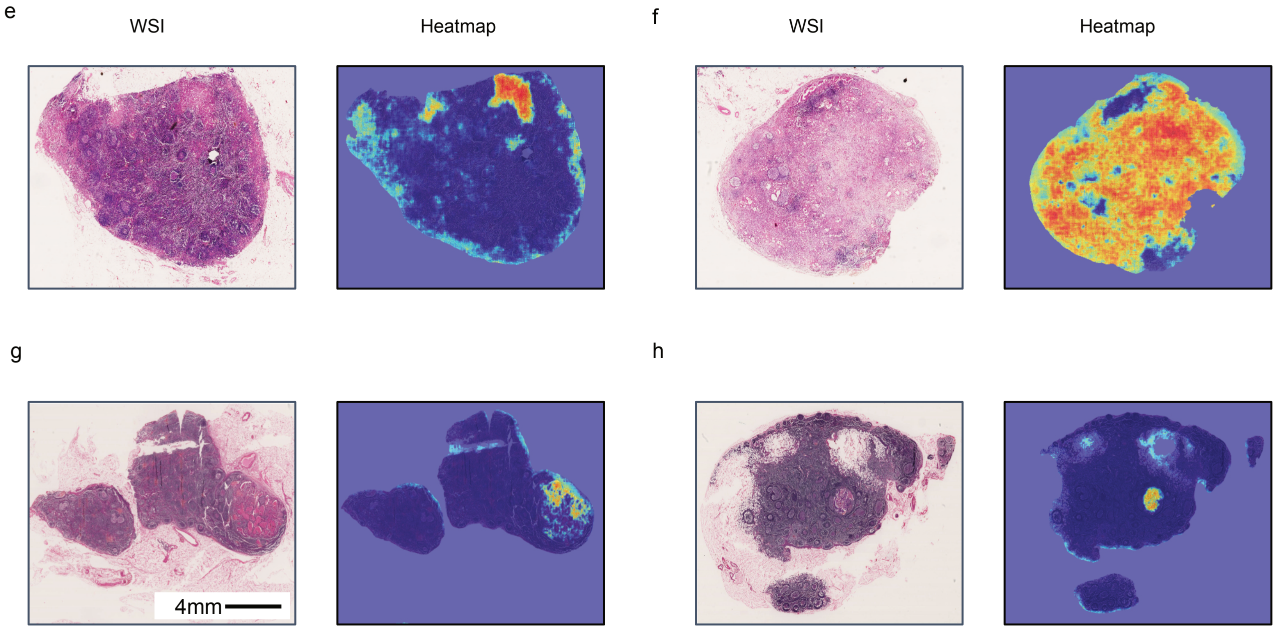

Supplementary Fig. 5. **Results of deep-learning framework performance analysis visualization of prediction results of the gastric cancer lymph nodes from the CH 2006~2008 and JX 2016~2019 GC cohorts**. (a, b) Comparison MLN of only AI and AI-assisted diagnosis with original diagnosis of the pathologist in CH 2006~2008 cohort (n=4,343 lymph nodes). (c, d) Comparison MLN of only AI and AI-assisted diagnosis with original diagnosis of the pathologist in JX 2016~2019 cohort (n=2,260 lymph nodes). We performed tumor detection in the gastric cancer lymph nodes area of the WSIs to generate superimposed heatmap. The redder the color, the higher the confidence of the tumor. (e, f) CH 2006~2008 GC lymph nodes. (g, h) JX 2016~2019 GC lymph nodes.

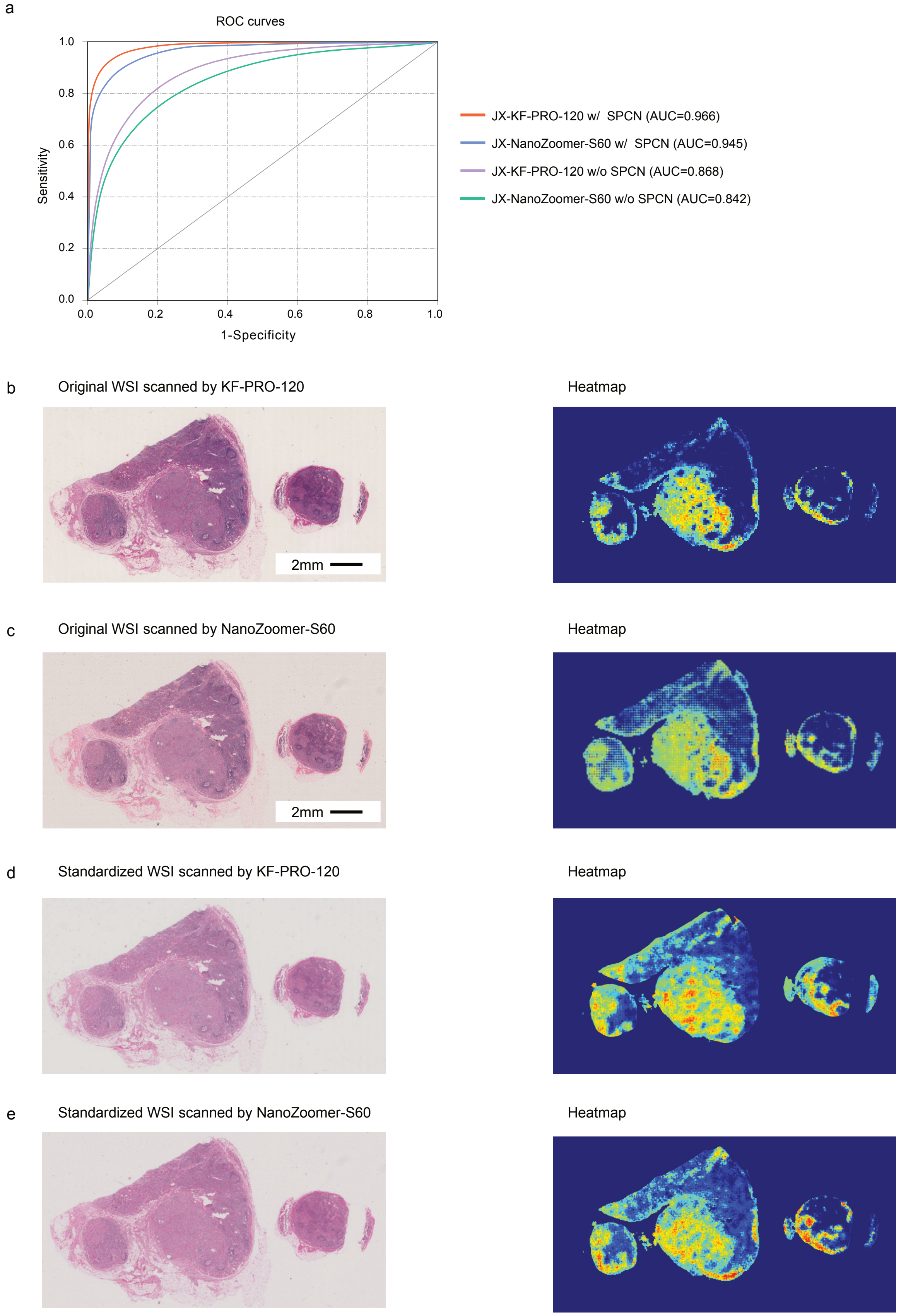

Supplementary Fig. 6. **Visualization of prediction results of the gastric cancer lymph nodes from the JX 2016~2019 GC cohort with different scanner.** We Compared the difference between the imaging of different scanners and the prediction results of the same classification network. (a) ROC curves of the system with and without SPCN for different scanner. (b) Original WSI scanned by KF-PRO-120. (c) Original WSI scanned by NanoZoomer-S60. (d) Standardized WSI scanned by KF-PRO-120. (e) Standardized WSI scanned by NanoZoomer-S60.

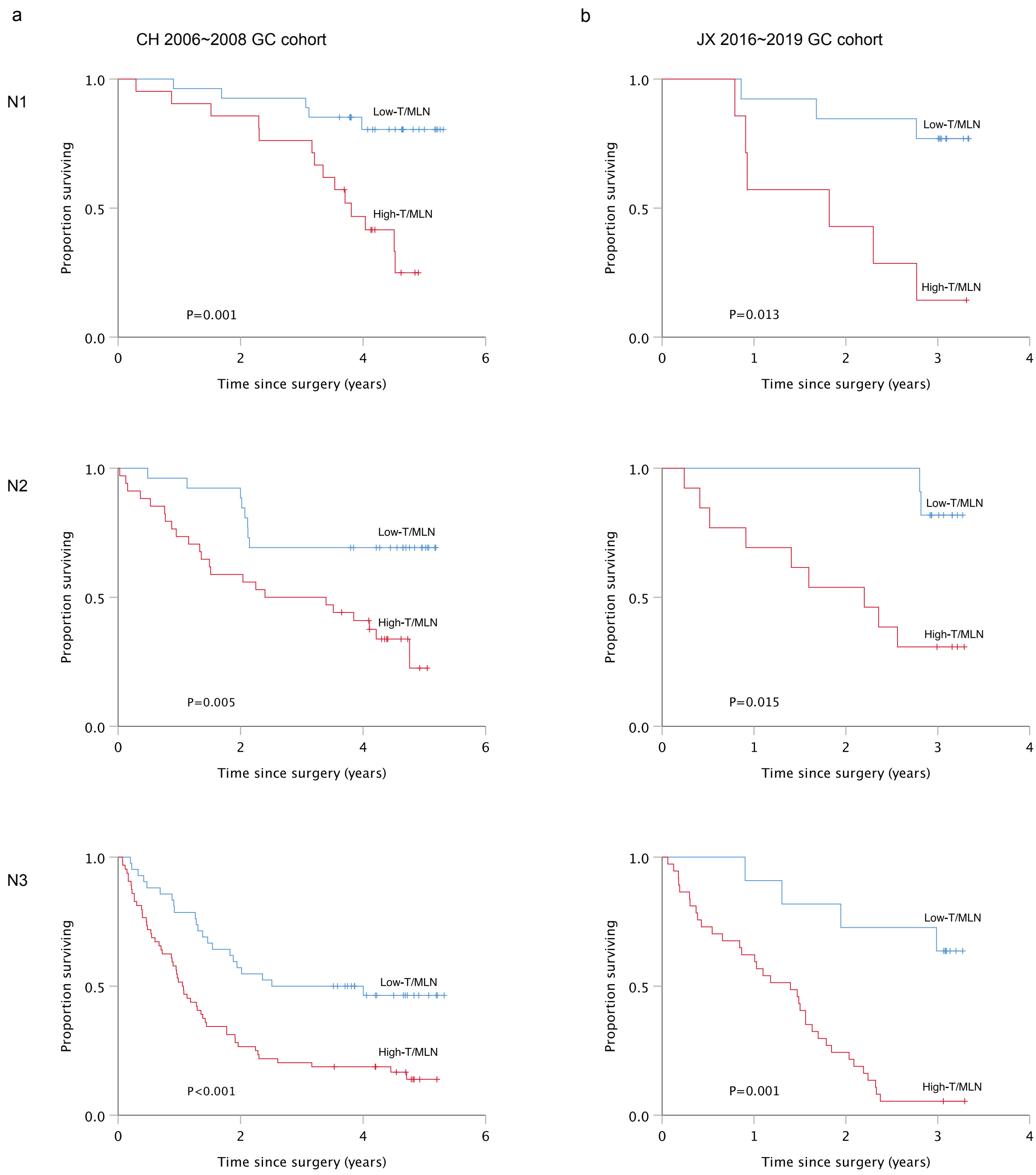

Supplementary Fig. 7. **Kaplan-Meier analysis of cancer-specific survival of T/MLN in the N-stage with low-T/MLN and high-T/MLN at the CH Hospital 2006~2008 GC cohort and JX Hospital 2016~2019 GC cohort.** (a) CH Hospital 2006~2008 GC cohort. (b) JX Hospital 2016~2019 cohort. *P* values were determined by two-sided log-rank test.

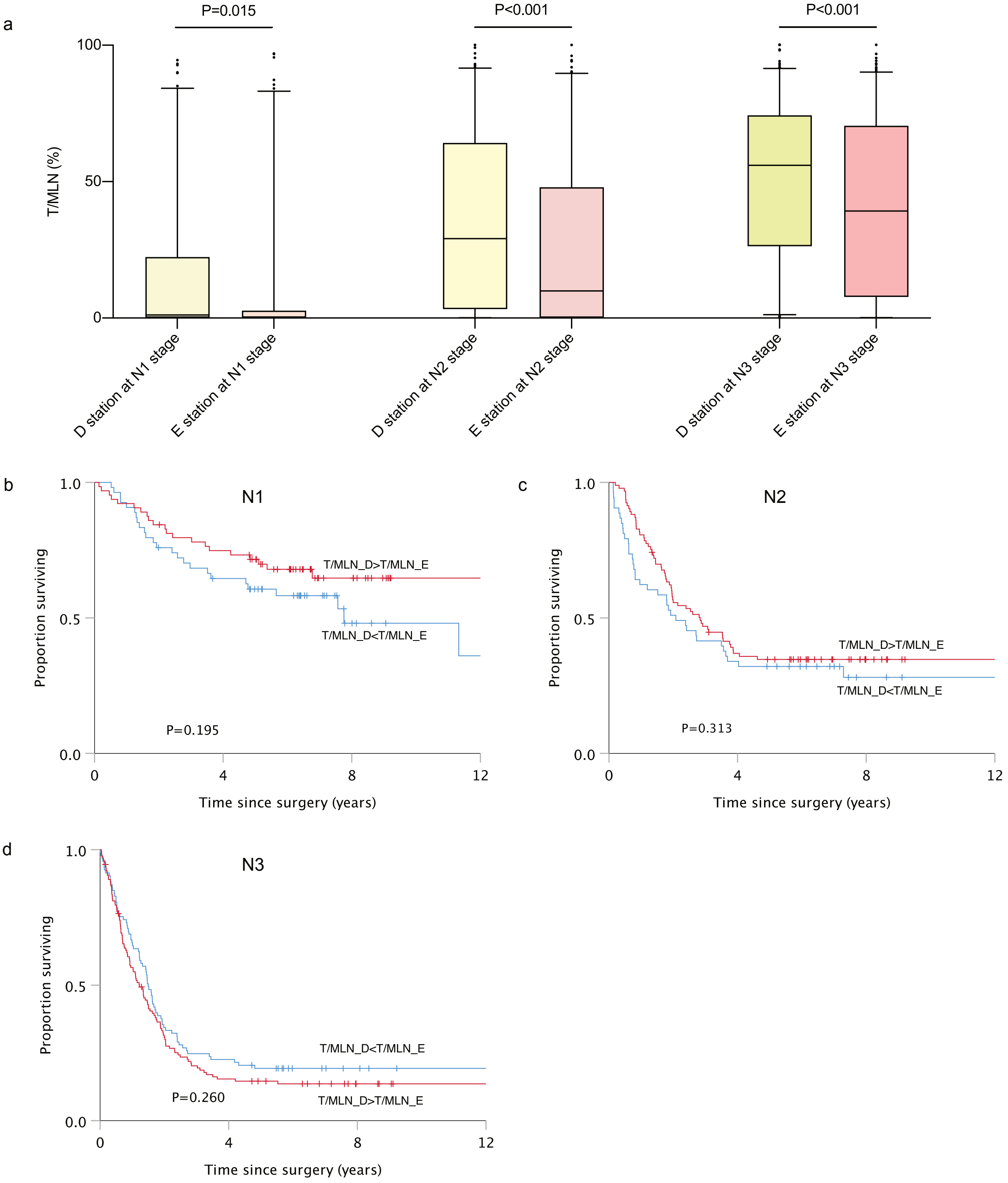

Supplementary Fig. 8. **Distribution statistics of T/MLN in station D and station E and KM analysis of cancer-specific survival in the N-stage with difference between D station's and E station's T/MLN at the CH Hospital 2001~2005 GC cohort.** (a) The distribution of T/MLN in station D and station E in the N-stage (N1 stage: n=127 patients; N2 stage: n=153 patients; N3 stage: n=236 patients). (b) The KM curve at N1 stage. (c) The KM curve at N2 stage. (d) The KM curve at N3 stage. For the boxplots, whiskers represent the ranges. The middle line within the box represents the median. The upper and lower boundaries of the whiskers represent the 5th and 95th percentiles, respectively. *P* values were determined by two-sided Wilcoxon matched-pairs signed rank test (a) or two-sided log-rank test (b-d).

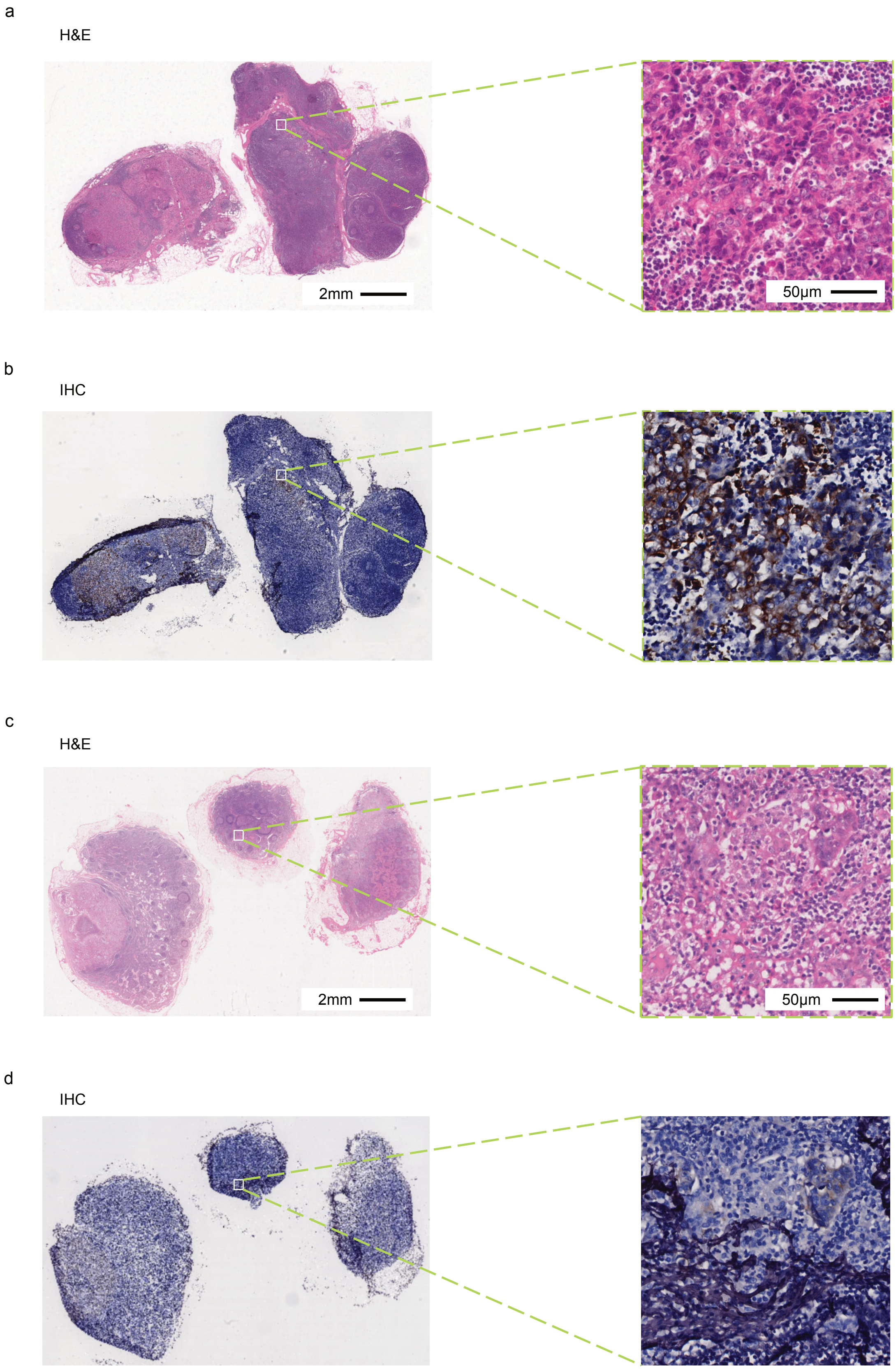

Supplementary Fig. 9. **Visualization of H&E staining and immunohistochemical restaining examples.** We used IHC (CAM5.2, MAB-0687, Fuzhou Maixin Biotech. Co., Ltd) restaining to resolve the slides of diagnostic uncertainty. (a, c) H&E stained WSI. (b, d) Immunohistochemical (IHC) restained WSI.

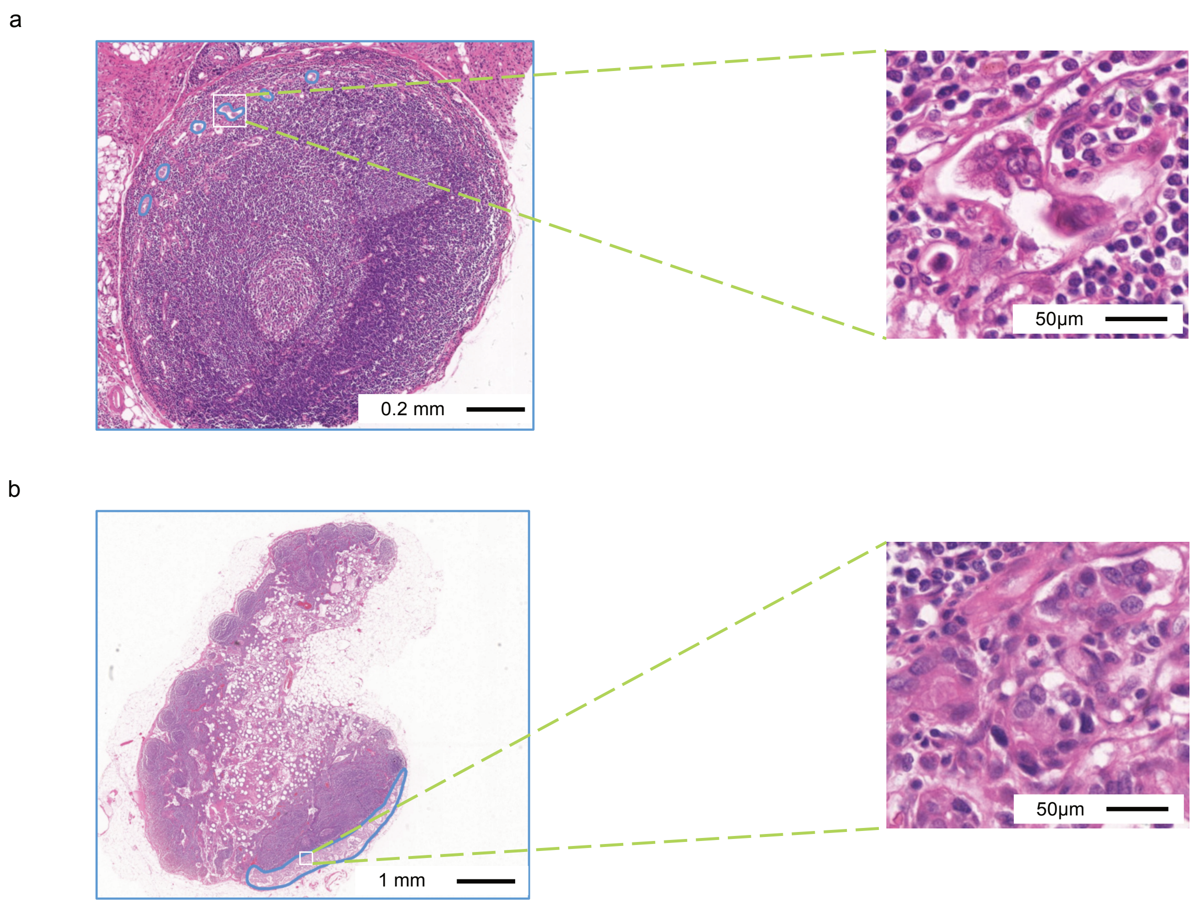

Supplementary Fig. 10. **Visualization of H&E stained lymph node with metastasis.** The regions outlined by the blue lines are the tumor. (a) In this example, there are multiple isolated tumor cells in the lymph node, the cumulative diameter is less than 2mm, and the calculation is cumbersome. (b) In this example, although the diameter of the metastasis in the lymph node is very long (about 15mm), it is relatively narrow, and the ratio of tumors to the lymph node is small (about 5%).

Supplementary Table 1. Baseline characteristics of patients with gastric cancer in CH Hospital 2001~2005 cohort and 2006~2008 cohort and JX Hospital 2016~2019 cohort for prognostic analysis

	CH2001-2005 GC	CH 2006-2008 GC	JX 2016-2019 GC
Number of patients	516	215	92
Follow-up time, years	7.6 (3.6~16.1)	4.5 (3.5~5.3)	1.68 (0.5~3.0)
Age at surgery, years	59 (23-86)	60 (27-86)	62(35-89)
≤60	245 (47.5%)	110 (51.1%)	39(42.4%)
>60	271 (52.5%)	105 (48.8%)	53(57.6%)
Sex			
Female	174 (33.7%)	41 (19.0%)	36(39.1%)
Male	342 (66.3%)	174 (80.9%)	56(60.9%)
Histological classification			
Adenocarcinoma	456 (88.4%)	192 (89.3%)	80 (87.0%)
Mucinous adenocarcinoma	44 (8.5%)	12 (5.6%)	3 (3.2%)
Signet cell carcinoma	13 (2.5%)	4 (1.9%)	1 (1.1%)
Squamous cell carcinoma	1 (0.2%)	4 (1.9%)	0 (0.0%)
Adeno-squamous cell carcinoma	0 (0.2%)	3 (1.4%)	2 (2.2%)
Undifferentiated carcinoma	2 (0.4%)	0 (0.0%)	0 (0.0%)
Lauren classification			
Intestinal	174 (33.7%)	66 (30.7%)	45 (48.9%)
Diffuse	121 (23.4%)	83 (38.6%)	31 (33.7%)
Mixed	220 (42.6%)	62 (28.8%)	16 (17.4%)
Undefined	1 (0.2%)	4 (1.9%)	0 (0.0%)
N stage			
N1	127 (24.6%)	48 (22.3%)	20(21.7%)
N2	153 (29.7%)	60 (27.9%)	24(26.1%)
N3	236 (45.7%)	107 (49.7%)	48(52.2%)
Pathological tumor (T) stage			
T1	29 (5.6%)	9 (4.18%)	4(4.3%)
T2	67 (13.0%)	18 (8.37%)	5(5.4%)
T3	372 (72.1%)	155 (72.0%)	8(8.7%)
T4	48 (9.3%)	33 (15.3%)	75(81.5%)
Size			
≤5	300 (58.1%)	120 (55.8%)	15(16.3%)
>5	216 (41.9%)	95 (44.1%)	77(83.7%)
Histological grade			
1	23 (4.5%)	2 (0.930%)	7(7.6%)
2	251 (48.6%)	93 (43.2%)	38(41.3%)
3	242 (46.9%)	120 (55.8%)	47(51.1%)
Surgery type			
Radical	411 (79.7%)	180 (83.7%)	71(77.2%)
Palliative care	105 (20.3%)	35 (16.2%)	21(22.8%)
Blood transfusion			
Yes	289 (56.0%)	113 (52.5%)	43(46.7%)
No	227 (44.0%)	102 (47.4%)	49(53.3%)
Location			
Cardia	64 (12.4%)	59 (27.4%)	19(20.7%)
Whole stomach	48 (9.3%)	14 (6.51%)	5(5.4%)
Gastric body	128 (24.8%)	41 (19.0%)	24(26.1%)
Pylorus	276 (53.5%)	101 (46.9%)	44(47.8%)

NOS, not otherwise specified; CH, Changhai; JX, Jiangxi Cancer; GC, gastric cancer.

Supplementary Table 2. Univariate and multivariate cancer-specific survival analysis of CH Hospital 2006~2008 gastric cancer cohort

	Univariable			Multivariable		
Variable	HR	95% CI	*P* value	HR	95% CI	*P* value
T/MLN (1: ≤0.45, 2: >0.45)	3.01	2.04,4.46	<0.001	2.92	1.82,4.70	<0.001
N stage (1-3: N1-N3)	1.8	1.42,2.30	<0.001	1.89	1.39,2.57	<0.001
Pathological tumor (T) stage (1-4: T1-T4)	1.35	1.01,1.81	0.046	0.79	0.53,1.18	0.245
Size (1: ≤5, 2: >5)	1.27	0.89,1.80	0.187	-	-	-
Histological grade (1-3)	0.84	0.59,1.18	0.308	-	-	-
Surgery type (1: radical, 2: palliative care)	3.21	2.03,5.08	<0.001	4.73	2.53,8.85	<0.001
Age at surgery, years (1: ≤60, 2:>60)	1.44	1.01,2.04	0.042	0.95	0.61,1.50	0.832
Sex (1: Male, 2: Female)	0.74	0.46,1.19	0.209	-	-	-
Histological type (1: Adenocarcinoma, 2: Other)	1.37	0.75,2.48	0.304	-	-	-
Lauren type (1: Intestinal, 2: Diffuse or mixed)	1.157	0.79,1,70	0.460	-	-	-
Blood transfusion (1: No, 2: Yes)	1.13	0.74,1.71	0.583	-	-	-
Location_pylorus	-	-	0.503	-	-	-
Location_cardia	1.06	0.71,1.59	0.775	-	-	-
Location_whole stomach	0.67	0.31,1.47	0.32	-	-	-
Location_gastric body	0.77	0.46,1.28	0.31	-	-	-

P values were determined by two-sided log-rank test. T/MLN, ratio of tumor area to metastatic lymph node area; HR, hazard ratio; CI, confidence interval.
