# Supplementary material for: Predicting gastric cancer outcome from resected lymph node histopathology images using deep learning
Source: Nat Commun. 2021 Mar 12;12:1637. doi: 10.1038/s41467-021-21674-7 (PMC7954798; doi:10.1038/s41467-021-21674-7)
Supplement: Supplementary file 2 — Reporting Summary [file 41467_2021_21674_MOESM2_ESM.pdf]

nature research

Corresponding author(s): Xiyang Liu and Guanzhen Yu

Last updated by author(s): Nov 29, 2020

Reporting Summary

Nature Research wishes to improve the reproducibility of the work that we publish. This form provides structure for consistency and transparency in reporting. For further information on Nature Research policies, see Authors & Referees and the Editorial Policy Checklist .

Statistics

For all statistical analyses, confirm that the following items are present in the figure legend, table legend, main text, or Methods section.

n/a	Confirmed	
☐	☒	The exact sample size (*n*) for each experimental group/condition, given as a discrete number and unit of measurement
☐	☒	A statement on whether measurements were taken from distinct samples or whether the same sample was measured repeatedly
☐	☒	The statistical test(s) used AND whether they are one- or two-sided<br>*Only common tests should be described solely by name; describe more complex techniques in the Methods section.*
☒	☐	A description of all covariates tested
☒	☐	A description of any assumptions or corrections, such as tests of normality and adjustment for multiple comparisons
☐	☒	A full description of the statistical parameters including central tendency (e.g. means) or other basic estimates (e.g. regression coefficient) AND variation (e.g. standard deviation) or associated estimates of uncertainty (e.g. confidence intervals)
☐	☒	For null hypothesis testing, the test statistic (e.g. *F*, *t*, *r*) with confidence intervals, effect sizes, degrees of freedom and *P* value noted<br>*Give P values as exact values whenever suitable.*
☒	☐	For Bayesian analysis, information on the choice of priors and Markov chain Monte Carlo settings
☒	☐	For hierarchical and complex designs, identification of the appropriate level for tests and full reporting of outcomes
☒	☐	Estimates of effect sizes (e.g. Cohen's *d*, Pearson's *r*), indicating how they were calculated

Our web collection on statistics for biologists contains articles on many of the points above.

Software and code

Policy information about availability of computer code

Data collection	Glass slides were digitized at 40x (0.25 µm/pixel) with Konfoong Bioinformation KF-PRO-120 scanner and (0.22 µm/pixel) NanoZoomer-S60.
Data analysis	The algorithms were written in python.<br>We used Openslide (version 1.1) to access the whole slide images, and Pytorch (version 1.3) to train deep learning models.<br>SPSS 25.0 was used for survival analysis.<br>Scikit-learn (version 0.21) was used to calculate the AUC.<br>Custom code related to the deep-learning model is available at https://github.com/wangxiaodong1021/auto_lymph.

For manuscripts utilizing custom algorithms or software that are central to the research but not yet described in published literature, software must be made available to editors/reviewers. We strongly encourage code deposition in a community repository (e.g. GitHub). See the Nature Research guidelines for submitting code & software for further information.

Data

Policy information about availability of data

All manuscripts must include a data availability statement. This statement should provide the following information, where applicable:

- Accession codes, unique identifiers, or web links for publicly available datasets
- A list of figures that have associated raw data
- A description of any restrictions on data availability

The datasets used to train models in this study will be publicly available. The test sub-dataset is available at https://doi.org/10.6084/m9.figshare.13065986. The complete datasets from Jiangxi Hospital and Changhai Hospital are not publicly available, because they contain protected patient privacy information. Source data are provided with this paper.

Field-specific reporting

Please select the one below that is the best fit for your research. If you are not sure, read the appropriate sections before making your selection.

- [x] Life sciences
- [ ] Behavioural & social sciences
- [ ] Ecological, evolutionary & environmental sciences

For a reference copy of the document with all sections, see nature.com/documents/nr-reporting-summary-flat.pdf

Life sciences study design

All studies must disclose on these points even when the disclosure is negative.

Sample size	The sample size was determined by the number of slides we cloud digitize in the two hospitals. The data set consists of four queues (Figure 1A). A total of 9,366 WSIs were generated from these groups, of which 21,965 were lymph nodes. Among them, 7,736 lymph nodes had metastatic lesions, and 14,229 lymph nodes had no metastases. Three of the cohorts were gastric cancer lymph nodes: one from 2001 to 2005 in the CH hospital (15,362), one from 2006 to 2008 in the CH hospital (4,343), and one from the JX hospital (2,260). The training sample set is from the dataset of CH 2001-2005. we selected 120 WSIs with tumor metastasis and 60 WSIs without tumor metastasis each year for labeling to improve network robustness and avoid bias. The datasets used in this research covers various subtypes of gastric cancer, sufficient for the models training and testing.
Data exclusions	We only included patients with malignant tumor of epithelial origin. Patients treated with neoadjuvant therapy were excluded. Only these with a total number of resected lymph nodes over 7 and good quality were enrolled in this study. Therefore, two of the cases from CH Hospital 2001-2005 were excluded from the analysis of the classification network. Due to the lack of prognostic information from patients in the CH hospital from 2001 to 2005, 341 patients were excluded from the prognostic analysis.
Replication	We tested the performance of the deep-learning framework and the prognostic predictions using three independent cohorts without retraining the networks. All attempts at replication were successful.
Randomization	We randomly selected 500 WSIs with tumor metastasis and 200 WSIs without tumor metastasis from this labeled sample set for network training, and the remaining 200 WSIs were used to verify network performance.
Blinding	These experiments of our deep learning models are based on digitized pathology slides. Two senior pathologists independently reviewed to generate the reference standard. A third pathologist was asked to help identify inconsistent results. If in doubt, we used IHC to confirm the final result. During the whole process, there is no need to know any clinical information about the patient. Therefore, there is no subjective bias in each pathologist review.<br>Clinical records are only used for retrospective prognostic analysis. There is no subjective bias here, so there is no need blinding.

Reporting for specific materials, systems and methods

We require information from authors about some types of materials, experimental systems and methods used in many studies. Here, indicate whether each material, system or method listed is relevant to your study. If you are not sure if a list item applies to your research, read the appropriate section before selecting a response.

Materials & experimental systems

n/a	Involved in the study	
x		Antibodies
x		Eukaryotic cell lines
x		Palaeontology
x		Animals and other organisms
	x	Human research participants
x		Clinical data

Methods

n/a	Involved in the study	
x		ChIP-seq
x		Flow cytometry
x		MRI-based neuroimaging

Human research participants

Policy information about studies involving human research participants

Population characteristics	Our dataset includes patients with gastric cancer from multiple centers. Among them, the detailed information of patients with gastric cancer is shown in Supplementary Table 1.
Recruitment	No patients were recruited. Analyze all digital images available during pre-established collections.
Ethics oversight	All patients in this study signed an informed consent form before the operation, which contained a statement on the pathological tissue and clinical data for clinical research. And, this study was approved by the ethics committees of the Changhai Hospital and Jiangxi Cancer Hospital.

Note that full information on the approval of the study protocol must also be provided in the manuscript.
